# Isolation and Characterization of the First Xylanolytic Hyperthermophilic Euryarchaeon *Thermococcus* sp. Strain 2319x1 and Its Unusual Multidomain Glycosidase

**DOI:** 10.3389/fmicb.2016.00552

**Published:** 2016-05-03

**Authors:** Sergey N. Gavrilov, Christina Stracke, Kenneth Jensen, Peter Menzel, Verena Kallnik, Alexei Slesarev, Tatyana Sokolova, Kseniya Zayulina, Christopher Bräsen, Elizaveta A. Bonch-Osmolovskaya, Xu Peng, Ilya V. Kublanov, Bettina Siebers

**Affiliations:** ^1^Winogradsky Institute of Microbiology, Research Center of Biotechnology, Russian Academy of SciencesMoscow, Russia; ^2^Molecular Enzyme Technology and Biochemistry, Biofilm Centre, Centre for Water and Environmental Research, University Duisburg-EssenEssen, Germany; ^3^Novozymes A/SBagsværd, Denmark; ^4^Department of Biology, University of CopenhagenCopenhagen, Denmark; ^5^Fidelity Systems, Inc., GaithersburgMD, USA

**Keywords:** archaea, hyperthermophiles, *Thermococcus*, xylan, multidomain glycosidase, endoglucanase

## Abstract

Enzymes from (hyper)thermophiles “Thermozymes” offer a great potential for biotechnological applications. Thermophilic adaptation does not only provide stability toward high temperature but is also often accompanied by a higher resistance to other harsh physicochemical conditions, which are also frequently employed in industrial processes, such as the presence of, e.g., denaturing agents as well as low or high pH of the medium. In order to find new thermostable, xylan degrading hydrolases with potential for biotechnological application we used an *in situ* enrichment strategy incubating Hungate tubes with xylan as the energy substrate in a hot vent located in the tidal zone of Kunashir Island (Kuril archipelago). Using this approach a hyperthermophilic euryarchaeon, designated *Thermococcus* sp. strain 2319x1, growing on xylan as sole energy and carbon source was isolated. The organism grows optimally at 85°C and pH 7.0 on a variety of natural polysaccharides including xylan, carboxymethyl cellulose (CMC), amorphous cellulose (AMC), xyloglucan, and chitin. The protein fraction extracted from the cells surface with Tween 80 exhibited endoxylanase, endoglucanase and xyloglucanase activities. The genome of *Thermococcus* sp. strain 2319x1 was sequenced and assembled into one circular chromosome. Within the newly sequenced genome, a gene, encoding a novel type of glycosidase (143 kDa) with a unique five-domain structure, was identified. It consists of three glycoside hydrolase (GH) domains and two carbohydrate-binding modules (CBM) with the domain order GH5-12-12-CBM2-2 (N- to C-terminal direction). The full length protein, as well as truncated versions, were heterologously expressed in *Escherichia coli* and their activity was analyzed. The full length multidomain glycosidase (MDG) was able to hydrolyze various polysaccharides, with the highest activity for barley β-glucan (β- 1,3/1,4-glucoside), followed by that for CMC (β-1,4-glucoside), cellooligosaccharides and galactomannan. The results reported here indicate that the modular MDG structure with multiple glycosidase and carbohydrate-binding domains not only extends the substrate spectrum, but also seems to allow the degradation of partially soluble and insoluble polymers in a processive manner. This report highlights the great potential in a multi-pronged approach consisting of a combined *in situ* enrichment, (comparative) genomics, and biochemistry strategy for the screening for novel enzymes of biotechnological relevance.

## Introduction

The use of molecular ecology approaches revealed that the vast majority of the total microbial biodiversity was so far not cultivated (Barns et al., 1996; Hugenholtz et al., 1998) highlighting that most of the natural diversity with an inestimable metabolic variability and potential is still hidden. This offers a great reservoir for novel biocatalysts with significant potential in biotechnological applications and process optimization. Enzymes from hyperthermophiles are typically folded into very stable conformations able to withstand high temperatures. The temperature stability is often associated with a high resistance to chemical denaturants commonly used in many industrial applications. The high stability combined with an optimal activity at high temperatures has led to a strong interest in using enzymes from hyperthermophiles in a wide range of commercial applications, most well-known, e.g., in starch and the cellulosic ethanol industries. Both of these applications require enzymes active at high temperatures, thus allowing better substrate solubility, easier mixing and lowered risk of contamination. Archaea are abundant in extreme habitats, especially in hyperthermophilic environments. They exhibit unique metabolic features and modified metabolic pathways characterized by unusual enzymes, not homologous to their bacterial counterparts. Therefore, archaeal enzymes offer great potential as novel biocatalysts for industry, which has continuously high demand for innovative solutions in the biocatalytic sector. Especially, (hyper)thermostable and thermoactive hydrolases degrading recalcitrant polysaccharides like cellulose and xylan into their monomeric constituents are of great importance for, e.g., pulp, biofuel and food industries (Egorova and Antranikian, 2005; Unsworth et al., 2007).

The majority of so far cultured hyperthermophilic organotrophic archaea of the two well-represented phyla, Euryarchaeota and Crenarchaeota are anaerobes growing by fermentation of various complex peptides and proteinaceous substrates, such as peptone, tryptone, beef, and yeast extract (Amend and Shock, 2001). In contrast, only few of these organisms were found to be able to grow on polysaccharides (**Supplementary Figure S1**). According to our knowledge, there are only three reports showing weak growth of hyperthermophilic archaea on cellulose and its derivatives, i.e., for *Desulfurococcus fermentans* (Perevalova et al., 2005), *Thermococcus sibiricus* (Mardanov et al., 2009) and a consortium of three species with predominance of an *Ignisphaera* representative (Graham et al., 2011). The latter was also shown to possess cellulase activity. Growth on xylan or heat treated xylan (121°C, 20 min) was demonstrated only for members of the *Crenarchaeota*, i.e., *Thermosphaera aggregans* (Huber et al., 1998), *Sulfolobus solfataricus* (Cannio et al., 2004), and *Acidilobus saccharovorans* (Prokofeva et al., 2009). In contrast to these scarce reports for growth of hyperthermophilic archaea on polysaccharides, genomes of many of these organisms harbor genes encoding glycoside hydrolases (GHs^1^, **Supplementary Table S1**), and several cellulases and xylanases were isolated from archaeal strains. However, most of these strains were either unable to grow on crystalline cellulose or xylan or were not analyzed for the ability to grow on these substrates (Ando et al., 2002; Cannio et al., 2004; Maurelli et al., 2008). Therefore, the function and efficiency of these enzymes for *in vivo* polymer degradation is still unclear.

For the identification of novel enzymes two main approaches are currently applied: They can be obtained either directly from the environment using high-throughput techniques such as (functional) metagenomics (Ferrer et al., 2015), or through the enrichment and isolation of novel microorganisms. In order to discover efficient biocatalysts, the isolation of novel strains with the desired properties, like the ability to cleave and to grow on cellulose or xylan is advantageous. Therefore, improved cultivation approaches have to be applied, such as providing the most environmentally close conditions for cultivation (Kublanov et al., 2009), utilization of novel substrates and/or electron acceptors, presence or absence of growth factors, as well as the inhibition of cultured fast-growing microorganisms.

Here we describe a multilayered approach for the isolation of novel biocatalysts for biotechnological applications using (i) an *in situ* enrichment strategy for organisms, that are capable of polymer degradation, (ii) genomics, (iii) comparative genomics as well as (iv) cloning and biochemical characterization of enzymes of interest. Using this *in situ* enrichment technique on mineral medium with xylan as the sole carbon source, we isolated a new representative of the *Euryarchaeota, Thermococcus* sp. strain 2319x1. The strain was able to grow on xylan, xyloglucan, alginate, amorphous and CMCs, starch and its derivatives, as well as on mono- and disaccharides. The complete genome of the novel strain was sequenced and revealed the presence of genes encoding diverse hydrolytic enzymes. One of these hydrolases, constitutes a novel multidomain enzyme with a unique three catalytic glycosidase and two carbohydrate-binding domain organization, called multidomain glycosidase (MDG). Upon expression in *Escherichia coli*, the purified recombinant enzyme (full-sized and truncated versions) exhibited endoglucanase as well as numerous additional activities. The broad activity spectrum appears to be facilitated by the modular multidomain architecture also allowing to processively degrade partially soluble and insoluble polymers.

## Materials and Methods

### Chemicals (for Growth Experiments and Enzyme Assays)

Starch was purchased from Merck (Germany). Yeast extract, peptone, gelatin, mono- and disaccharides, barley glucan, birchwood and beechwood xylan, carboxymethyl cellulose (CMC), microcrystalline cellulose (MCC) Avicel, inulin, cellobiose, dextrin, dextran, pullulan, laminarin, lichenan, pectin, and alginate were purchased from Sigma Aldrich (Taufkirchen, Germany), or kindly provided by Dr. R. Wohlgemuth. Agarose (agarose MP) was purchased from Boehringer (Mannheim, Germany) and chitin (crab chitin) from Bioprogress (Russia). Chitin and chitosan were kindly provided by Dr. S. Lopatin from the Centre of Bioengineering, Research Center of Biotechnology, RAS, Moscow, Russia. Amorphous chitin (AMCH) and amorphous cellulose (AMC) for growth experiments and native activity measurements were prepared according to Sorokin et al. (2015). Other polysaccharides, such as glucomannan, galactomannan, arabinoxylan, and curdlan, were purchased from Megazyme (Ireland). Bamboo leaves collected near the sampling site were dried at room temperature and used as the growth substrate.

### Sampling, Enrichment, Isolation and Cultivation

Sampling and primary enrichment procedures were performed during the expedition to Kunashir Island (Russia) in July 2011. Hungate tubes (18 mL) containing 200-300 mg of CMC, chitin, agarose, or birchwood xylan, with or without amorphous Fe(III) oxide (ferrihydrite) as external electron acceptor were prepared. Tubes were filled with thermal water and sediments from the sampling site (coordinates: 44°11.08333′, 145°50.46667′), sealed with rubber stoppers and screw caps. Two 1.2-mm syringe needles were inserted into the rubber stoppers to allow the fluid exchange with the environment without a significant loss of insoluble substrates. The enrichment setups were placed into the hot spring, incubated for 6 days, and afterwards sealed and transferred to the lab at ambient temperature. A modified Pfennig medium with ferrihydrite (Gavrilov et al., 2007) or elemental sulfur (10 g L^-1^) was used for the isolation and metabolic characterization of new organisms. The medium contained 0.12 g L^-1^ Na_2_Sx9H_2_O, 9.0 g L^-1^ NaCl, 2.0 g L^-1^, MgCl_2_x6H_2_O, and 0.05 g L^-1^ of yeast extract; the pH was adjusted to 6.0–7.0. Ten mililiter portions of the medium were dispensed in 18 mL Hungate tubes with 10 mg birchwood xylan or with other substrates as carbon and energy source as indicated. Tubes were inoculated with 1/10 (v/v) of primary enrichments and incubated at temperatures close to values at respective sampling sites. Pure cultures were obtained by the serial dilution-to-extinction technique in the same liquid media.

### DNA Isolation, Genome Sequencing and Annotation

The culture was centrifuged at 4500 × *g* for 10 min, and the cell pellet was resuspended in 1 mL TNE buffer, pH 7.4 (20 mM TRIS HCl, 15 mM NaCl, 20 mM EDTA). Three repeated freezing and thawing cycles followed by 30 min incubation at room temperature with the addition of lysozyme (200 μg mL^-1^) and RNase (DNAse-free, 5 μg mL^-1^) were performed to disrupt the cells. Subsequently, proteinase K (10 μg mL^-1^) and SDS [0.5% (w/v)] were added, and the mixture was incubated for 30 min at 54°C. DNA was extracted using phenol:chlorophorm:isoamyl alcohol (50:50:1), pelleted by centrifugation (17000 × *g*, 4°C), washed (70% ice-cold ethanol) and dissolved in TE buffer (10 mÌ TRIS, 1 mÌ EDTA, pH 7.4). DNA sequencing was performed by BGI at Shenzhen, China^2^. A 500 bp paired-end library was constructed and sequenced with Illumina HiSeq using the standard protocol of the company.

The Ilumina sequencing resulted in ∼6.1 million read pairs of 2 × 90 bp length, which equals to a ∼560-fold coverage given the final genome size of 1,961,221 bp. An assembly using Velvet (Zerbino and Birney, 2008) resulted in 20 contigs. This initial assembly was used to identify repeat regions that were subsequently removed. Because of the presence of multiple repeat regions longer than 500 bp, a 2 kb mate-paired Illumina library was constructed and sequenced, and the obtained paired end information was used to arrange multiple screened contigs into a single scaffold using the Phred/Phrap/Consed (Gordon et al., 1998) software package. This package was used for further sequence assembly and quality assessment in the subsequent finishing process. Sequence gaps between contigs that represented repeats were filled with Dupfinisher (Han and Chain, 2006), and a single scaffold was created and verified using available paired end information. Together, the combination of reads from the two Illumina libraries provided a 700-fold coverage of the genome. The origin of replication was predicted using the Orifinder2 tool (Luo et al., 2014). The initial *de novo* gene prediction and annotation was done using the RAST web server (Aziz et al., 2008), which employs the Glimmer-3 gene caller (Delcher et al., 2007). Additionally, non-coding RNAs were predicted by Infernal 1.1 (Nawrocki and Eddy, 2013) using Rfam 12.0 (Nawrocki et al., 2015) as reference. A further manual curation of the RAST functional annotation was accomplished according to Toshchakov et al. (2015).

### Native Enzyme Activity Measurements

Native enzyme activities were determined in culture broth as well as in protein fractions that were extracted from cell surfaces. The extraction was performed in 0.05 M MOPS, pH^25°C^ 8.3 with 0.5% (v/v) Tween-80, which appeared to be the most efficient among various tested compounds [i.e., 1 M NaCl, 3 M urea, 0.18 mM SDS, 0.5% (v/v) Triton X-100 or 0.5% (v/v) Tween-80] for solubilization of proteins anchored to the cell surface (as determined by liberation of proteins into the solution after 1 h at 25°C). The resulted protein solution was diluted 10-fold with 0.05 M MOPS pH^25°C^ 8.3. Glycosidase activities were determined using the dinitrosalicylic acid (DNS) assay (Miller, 1959). Optical density values were converted into sugar concentrations using a D-glucose calibration curve. The calibration curve with D-xylose showed a similar slope as with D-glucose. To ensure that β–glucan does not interfere with the assay, a β–glucan calibration curve was performed, which revealed no effect of β-glucan below a concentration of 0.05 g L^-1^, both at 25 and at 85°C. Protein concentrations were measured using the bicinchoninic method (Smith et al., 1985).

### Cloning of the *mdg* Gene and its Truncated Versions for Protein Expression in *E. coli*

The signal sequence of the *mdg* gene encoding the MDG (ADU37_CDS22600) was identified using the SignalP tool^3^. The cloning of the *mdg* gene (3912 bp) and its truncated versions was performed using the In-Fusion^®^HD Cloning Kit (Takara Bio Company). For PCR amplification primers with 15 bp extensions at the 5′ ends corresponding to the sequence of the *Not*I (forward) and *Eco*RI (reverse) linearized expression vector pET24a were designed for the *mdg* gene and all gene truncations (**Supplementary Table S2**).

PCR products were purified with the Wizard^®^SV Gel and PCR Clean-Up System (Promega). Plasmid DNA was isolated with the GeneJET Plasmid Miniprep Kit (Fermentas). The In-Fusion^®^cloning reaction was performed for 15 min at 50°C and the reaction mixtures were directly transformed into Stellar competent cells (Takara) following the instructions of the manufacturer. The presence of the *mdg* full length and truncated inserts was confirmed by digestion of the respective plasmid DNA with gene/fragment specific restriction enzymes (i.e., *Xba*I, *Hind*III, *Xho*I and *Eco*RI), followed by sequencing (LGC Genomics, Berlin).

### Protein Expression and Purification

Expression of the recombinant MDG (GH5-12-12-CBM2-2) and the different truncated proteins (GH5-12-12, GH5-12 or single GH5) was performed in *E. coli* BL21 (DE3)–CodonPlus-pRIL (Novagen). For large scale protein expression 2 L fresh LB medium containing 50 μg mL^-1^ kanamycin and 50 μg mL^-1^ chloramphenicol was inoculated with 20 mL of an overnight culture. The cells were grown at 37°C with constant shaking at 180 rpm. Protein expression was induced at OD(600 nm) 0.6–0.9 by addition of 1 mM IPTG (isopropyl-β-D-thiogalactopyranosid) and the cells were grown at 30°C overnight with constant shaking (180 rpm). The cells (∼12 g wet weight) were harvested by centrifugation (6000 × *g* for 15 min at 4°C) and either stored at -80°C or directly resuspended in 40 mL buffer (50 mM TRIS HCl pH^25°C^ 8.0) for further purification. Cell lysis was performed by passing the cells three times through a French pressure cell at 1200 psi in the presence of protease inhibitors (cOmplete ULTRA Tablets, Mini, EDTA-free, EASYpack, Roche). Cell debris and unbroken cells were removed by centrifugation (12000 × *g* for 30 min at 4°C) and the resulting crude extract was diluted 1:1 and subjected to a heat precipitation for 30 min at 60°C. After heat precipitation, the samples were cleared by centrifugation (12000 × *g* for 30 min at 4°C) and dialyzed overnight against 50 mM TRIS HCl pH^25°C^ 7.0. The partial purified protein fractions were either used immediately for enzyme assays (MDG, GH5-12-12 protein) or further purified (GH5 and GH5-12 proteins). Since an affinity chromatography by Ni-TED column revealed no binding of the His-tagged GH5 and GH5-12 proteins, an alternative purification protocol via fractionated ammonium sulfate precipitation, ion exchange chromatography and size exclusion chromatography was established (for detailed description see Supplementary section Purification of the GH5 and GH5-12 Protein).

### Visualization of Cellulase Activity on Substrate Agar Plates and Zymogram Gels

For the first qualitative analysis, cellulolytic activity was followed using substrate agar plates and zymogram gels. Substrate agar plates contained 1.5% (w/v) agar-agar and 0.2% (w/v) CMC. The protein samples (5–15 μg, after heat precipitation) were transferred into punched holes and the plates were incubated at 60°C for 4 h or overnight. For zymography 0.2% (w/v) CMC was embedded in standard 7.5% SDS-PAGE gels (10 × 7.5 cm). Enzyme aliquotes were added to sample buffer (100 mM TRIS HCl, pH^25°C^ 6.8, 10% (w/v) SDS, 5% (v/v) 2-mercaptoethanol, 1% (w/v) bromphenol blue) but were not heated before loading. In each lane 2–3 μg of recombinant protein was applied and as protein standard the PageRuler Prestained Protein Ladder (Thermo Scientific) was used. After electrophoreses (BioRAD Mini-Protean System) gels were incubated twice for 20 min in renaturation buffer (100 mL, 50 mM potassium phosphate buffer pH 6.5, 2.5% (v/v) Triton X-100) at room temperature followed by 30 min incubation in developing buffer (100 mL volume; 20 mM MOPS pH^25°C^ 6.5, 100 mM NaCl, 2 mM CaCl_2_). Finally, in order to visualize endoglucanase activity gels and plates were stained with 0.2% (w/v) Congo red for 30 min (5 mL for plates, 10 mL for gels) and destained with 1 M NaCl three times over 15 min at room temperature. Crude extract of *E. coli* BL21 (DE3)-CodonPlus-pRIL with empty vector was used as control for substrate agar plates and zymogram gels.

### Analysis of Hydrolysis Products by Thin Layer Chromatography (TLC)

Enzymatic reactions with 1% (w/v) oligomeric substrates (Megazyme) were performed under the same conditions as used for the DNS assay. Glucose, cellobiose, cellotriose, cellotetraose, cellopentaose, and cellohexaose were incubated with the MDG and the three truncated enzymes (25 μg) at 60°C for 120 min. Controls (stored on ice) as well as hydrolysis products were separated on aluminum sheet (20 × 20 cm) silica gel 60/kieselguhr F_254_ plates (Merck) with ethyl acetate, methanol and H_2_O (68:23:9, v/v/v) as a solvent and visualized with KMnO_4_ solution (1.5 g KMnO_4_, 10 g K_2_CO_3_ and 1.25 mL 10% aq. NaOH in 200 mL H_2_O).

### Activity Measurements of Recombinant Enzymes

Enzyme activities were determined in the presence of soluble polysaccharide substrates, i.e., β-D-glucan from barley, CMC, hydroxyethyl cellulose (HEC), birchwood xylan and other β-linked polysaccharides. Standard assay mixtures contained 100 μL McIlvaine buffer (0.2 M Na_2_HPO_4_ titrated with 0.1 M citric acid pH^25°C^ 6.0) and 250 μL of the respective 1% (w/v) substrate solution. After pre-equilibration for 5 min at 60°C, the reaction was started by the addition of enzyme. Following a 30 min incubation at 60°C, the reaction mixture was transferred to ice for 5 min, mixed with 750 μL DNS reagent (Bernfeld, 1955), and boiled for 10 min at 100°C. The color shift from yellow to brown depends on the amount of reducing groups and was determined at 575 nm. One unit of enzyme activity was defined as the amount of enzyme required to release 1 μmol glucose equivalents per minute. The pH and temperature optimum for the full length MDG (GH5-12-12-CBM2-2) and the GH5 protein were determined with partially purified enzyme (44 μg) and purified protein (28 μg), respectively, using β-D-glucan [1% (w/v)] from barley as the substrate. AMC was produced by phosphoric acid treatment of Avicel as described previously by Zhang et al. (2006).

## Results

### Enrichment, Isolation and General Features

Strain 2319x1 was isolated from an *in situ* enrichment with birchwood xylan as the energy and carbon source, and Fe(III) (in the form of insoluble ferrihydrite) as the electron acceptor. The initial inoculum consisted of black sand (enriched with mixed valence Fe mineral magnetite) and hot water from a hot spring, located in the tidal zone near Goryachiy cape of Kunashir Island (South Kurils, Russian Far East region) (**Supplementary Figure S2**). The enrichment culture was incubated in the same spring, with temperature and pH fluctuating in the range of 76–99°C and 5.0–7.0, respectively. After 6 days of incubation the culture contained two major morphotypes: (i) short rods later on identified as *Pyrobaculum arsenaticum* (manuscript in preparation) and (ii) small irregular cocci. Reduction of ferrihydrite to a magnetic mineral was observed. After the next three subsequent transfers at 90°C on a modified Pfennig medium (pH 6.0–6.2) containing xylan and ferrihydrite but devoid of yeast extract, the coccoid cells became dominating, while the rods disappeared and ferrihydrite reduction ceased. When ferrihydrite (90 mM) was replaced with elemental sulfur (5 g L^-1)^, hydrogen sulfide was formed, but no detectable stimulation of growth was observed. After 10-fold serial (up to 10^-8^) dilutions on the initial medium with xylan and ferrihydrite, a pure culture was obtained designated as strain 2319x1. 16S rRNA gene-based phylogenetic analysis revealed *T. alcaliphlus*, *T. sibiricus*, and *T. litoralis* as the nearest validly published relatives (99.7, 98.4, and 99.5% of 16S rRNA gene identity, respectively; **Supplementary Figure S3**). All substrates supporting growth of strain 2319x1 in addition to yeast extract and cellulose as well as those which did not serve as growth substrates are depicted in **Table 1**. During the growth on xylan the generation time was ca. 1 h and the final growth yield was 2.5 × 10^7^ cells mL^-1^.

**Table 1:** Growth substrates of *Thermococcus* sp. strain 2319x1.

Utilized substrates	Non-utilized substrates
Yeast extract	D-mannose
Peptone	D-fructose
Gelatin	Cellobiose
D-xylose	Trehalose
D-glucose	Raffinose
Lactose	β-glucan
Maltose	Avicel
Sucrose	arabinoxylan
Dextrin	arabinan
Dextran	Arabinogalactan
Pullulan	Galactan
Starch	Galactomannan
Xylan	Glucomannan
Xyloglucan	Mannan
AMCH	
Chitosan	Laminarin
Amorphous cellulose (AMC)	Curdlan
CMC	Inulin
Alginate	Agarose
Lichenan	Pectin
Bamboo leaves	


### Genome Sequence and General Genome Features

The final, assembled circular chromosome comprises 1,961,221 bp with an average GC content of 44.6%. The RAST gene caller identified 2,294 protein coding genes. Eight hundred and sixty-four of these protein coding genes were designated as “hypothetical,” since no assigned database matches could be found. Both, RAST and Infernal identified the same set of the four rRNAs and 46 tRNAs. The rRNA genes are not located in a single operon: 16S and 23S rRNAs are separated by a single tRNA gene, while two non-identical (two nucleotide substitutions per molecule) 5S rRNAs are distantly located.

Additionally, the Infernal annotation revealed the presence of SRP RNA (signal recognition particle RNA) and RNAse P RNA, several riboswitches and the SscA RNA (secondary structure conserved A RNA), a putative non-coding RNA conserved in hyperthermophiles, which is so far exclusively found in the genera *Pyrococcus* and *Thermococcus.*

The average nucleotide identity (ANI) between the genomes of strain 2319x1 and *T. litoralis* (90.4%) or *T. sibiricus* (77.7%) is well below the species level of 95% (Goris et al., 2007). Together with 16S rRNA-based phylogenetic analysis, these results suggest that either strain 2319x1 belongs to the *T. alcaliphilus* species (so far no genome sequence is available) or represents a novel species of this genus.

The *Thermococcus* sp. strain 2319x1 genome is deposited in GenBank under the accession number CP012200.

### Genome-Scale Metabolic Reconstruction of Polysaccharide, D-Glucose and D-Xylose Degradation

**FIGURE 1:**
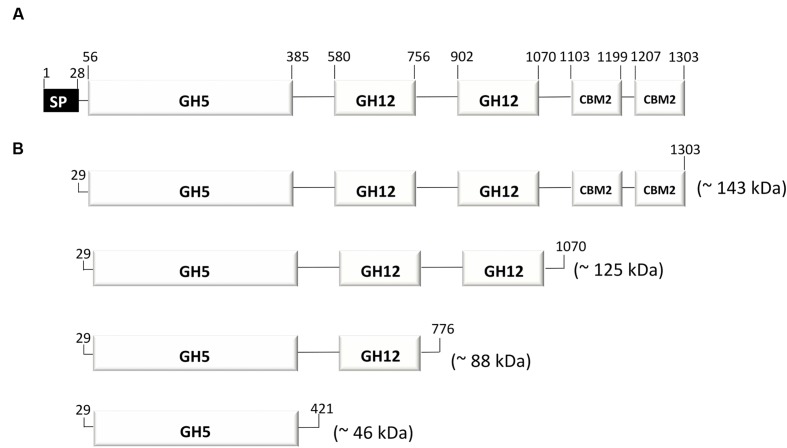
**The multidomain glycosidase (MDG) from *Thermococcus* sp. strain 2319x1.** The predicted sequence signatures and domain-architecture **(A)** and generated recombinant versions of the protein [full length MDG (GH5-12-12-CBM2-2), GH5-12-12, GH5-12 and single GH5] **(B)** are shown. SP, signal peptide; GH5, glycoside-hydrolase family No. 5; GH12, glycoside-hydrolase family No. 12; CBM2, cellulose binding-module family 2.

A total of 18 genes, encoding GHs and carbohydrate esterases (CEs) of different CAZy families (Lombard et al., 2014) were identified in the genome of strain 2319x1 (**Supplementary Table S3**). Four of them were predicted to be extracellular. Among those, only one protein had a C-terminal transmembrane region (ADU37_CDS18940, protein sequence region 1075–1097) presumably involved in anchoring the enzyme to the cell membrane. In agreement with the list of substrates supporting growth, the majority of glycosidases were annotated to be involved in hydrolysis of alpha-linked poly- and oligosaccharides (starch, dextrin, dextran, pullulan etc.), while only five enzymes were predicted to catalyze the hydrolysis of β-glycosides. The only exception appeared to be encoded by ADU37_CDS22600 which was predicted to be an endoglucanase/endoxylanase. The protein shows an unique domain organization and is composed of three GH domains (one GH family 5 and two GH family 12), and two family 2 carbohydrate-binding modules (CBM2), with the N- to C-terminal domain order GH5-12-12-CBM2-2 (**Figure 1A**). The analysis of signal peptide cleavage sites using SignalP (Petersen et al., 2011) and transmembrane helices using TMHMM (Möller et al., 2001) revealed an N-terminal signal peptide (amino acid residues 1–28).

Besides GHs and CEs, the genome encoded about 20 glycosyl transferases (GTs) of different families (mainly GT2 and GT4, according to CAZy). The majority is presumably involved in glycosylation of *S*-layer proteins, exopolysaccharide (biofilm) formation or biosynthesis of intracellular oligo- and/or polysaccharides. Few of them belong to families with a retaining mechanism, which theoretically allows either hydrolysis or phosphorolysis of glycosidic bonds. One of these proteins, encoded by ADU37_CDS05640 belongs to the GT35 family containing glycogen phosphorylases and enzymes with related activities.

Since strain 2319x1 was growing on various poly- and monosaccharides, it should possess an efficient uptake system for diverse mono-, di- and oligosaccharides. Indeed, more than 100 genes coding for transporters of the ATP-binding Cassette (ABC) Superfamily (3.A.1) were identified in the genome, along with over 20 genes coding for the Major Facilitator Superfamily (MFS, 2.A.1) transporters and two genes coding for the Solute:Sodium Symporter (SSS) family (2.A.21, according to TCDB database) (Saier et al., 2014).

The majority of substrates supporting the growth of the strain contained either D-glucose (D-glucose itself, maltose, starch and derivatives, cellulose and derivatives, xyloglucan) or D-xylose (D-xylose itself, xylan and xyloglucan). Accordingly, all genes encoding enzymes operative in a *Thermococcales* like modified Embden-Meyerhof-Parnas (EMP) pathway were identified (Siebers and Schönheit, 2005; Bräsen et al., 2014) (e.g., archaeal ADP glucokinase ADU37_CDS0192; ADP phosphofructokinase ADU37_CDS0945; glyceraldehyde-3-phosphate ferredoxin:oxidoreductase (GAPOR) ADU37_CDS1714; non-phosphory lating NAD(P)^+^- dependent glyceraldehyde-3-phosphate dehydrogenase (GAPN) ADU37_CDS1986). In contrast, the pathway for D-xylose degradation remained unclear. The only characterized archaeal D-xylose degradation pathway, described for *Haloferax volcanii* (Johnsen et al., 2009) and *S. solfataricus* (Nunn et al., 2010) seems to be absent since not a single protein of this pathway was found in the *in silico* translated proteome of strain 2319x1. Moreover, BLAST searches of key enzymes of this pathway did not reveal any homologs in other archaea, except haloarchaea and few members of the *Thermoprotei*. In bacteria, D-xylose can be utilized similarly to the *H. volcanii* pathway, as demonstrated, e.g., for *Caulobacter crescentus* (Stephens et al., 2007). Alternatively and more commonly, D-xylose degradation is initiated by xylose isomerase and kinase yielding xylulose-5-phosphate, which further enters the metabolic network via the pentose-phosphate pathway (Bräsen et al., 2014). However, no obvious homologs (e.g., xylose isomerase, xylulose kinase) involved in the established bacterial route for D-xylose degradation were identified. Therefore, the pathway for D-xylose degradation still remains unclear, the genome contains numerous genes encoding NAD(P)^+^-dependent oxidoreductases, sugar kinases as well as aldose-ketose isomerases with unknown or uncertain function, possibly involved in D-xylose degradation. The modified Entner–Doudoroff pathway (Bräsen et al., 2014) as well as the complete TCA cycle is absent, as it was shown for other members of *Thermococcales* (Atomi et al., 2005). Homologs for pyruvate:ferredoxin oxidoreductase and several ADP-forming ac(et)yl-CoA synthetases were identified in the genome of strain 2319x1, indicating a similar energy metabolism as described for *T. kodakarensis* and *P. furiosus* (Siebers and Schönheit, 2005; Bräsen et al., 2014).

Strain 2319x1, as most of other archaea, possesses no classical pentose-phosphate pathway (PPP). The genes, encoding proteins of the oxidative part of the PPP were absent, and only an incomplete set of proteins of the non-oxidative part was identified, i.e., a transketolase, which is split in an N- and C-terminal part (ADU37_CDS1012 and ADU37_CDS1013) and a ribose-5-phosphate isomerase (ADU37_CDS0756) (Bräsen et al., 2014). D-ribulose-5-phosphate is formed from D-fructose 6-phosphate via the reversed Ribulose Monophosphate Pathway – a two-step reaction, catalyzed by D-arabino-3-hexulose-6-phosphate formaldehyde-lyase and phosphohexuloisomerase (Kato et al., 2006; Bräsen et al., 2014). In *T. kodakaraensis* the genes, encoding these enzymes, are fused (Orita et al., 2006), and a highly similar homolog was found in the genome of strain 2319x1 (ADU37_CDS03570).

### Identification of Extracellular Polysaccharolytic Activities

**FIGURE 2:**
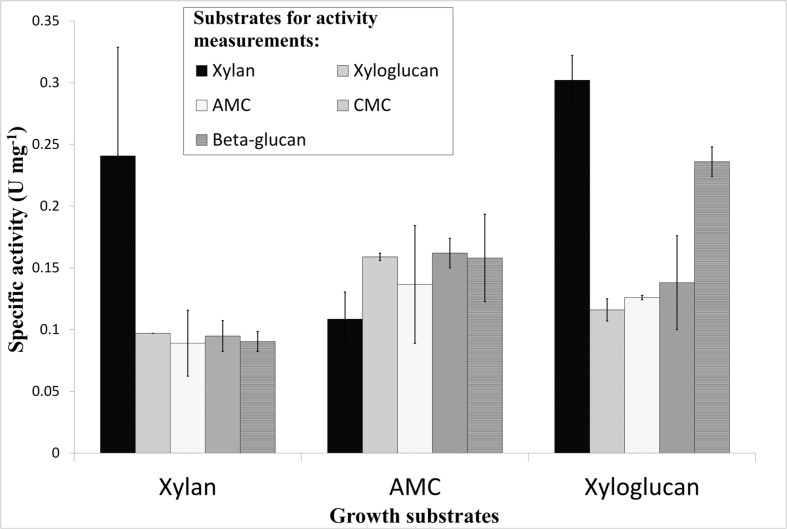
**Hydrolytic activities of surface protein fractions from strain 2319x1 cells, grown on xylan, AMC and xyloglucan.** The formation of reducing sugars using the DNS method per mg of protein (bicinhoninic assay) is shown. The different growth substrates (2 g L^-1^) (*x*-axis) as well as the substrates for activity measurements (0.01 g L^-1^) (different gray shades of columns shown in the right part of the figure) are depicted. Incubations were performed at 85°C for 20 h.

During the growth of strain 2319x1 on AMC, xylan and xyloglucan extracellular, cell-bound endoglucanases with broad specificity were produced (**Figure 2**). The cell yields were quite similar (1–1.5 × 10^8^ cells mL^-1^) for all three substrates. The concentrations of total cell-associated proteins were also of the same order of magnitude (49.6, 84.6, and 66.7 μg mL^-1^ for AMC, xylan or xyloglucan, respectively). In general, the determined activities in extracellular cell surface-bound protein fractions were not clearly linked to the different growth substrates utilized (**Figure 2**). This might be explained by the co-occurrence of cellulose and hemicelluloses in plant cell walls, resulting in the expression of the same enzyme or enzyme sets during the growth on both types of polysaccharides. It is important to mention that cells grown on gelatin did not produce detectable amounts of extracellular polysaccharide degrading enzymes (data not shown) further supporting their inducible state.

### Cloning, Expression and Purification of the Full Length MDG and Truncated MDG Proteins

Among the identified hydrolases, ADU37_CDS22600 was chosen for further analysis due to its unusual domain composition, GH5-12-12-CBM2-2. The *mdg* gene (3912 bp) encodes for a protein with a predicted molecular mass of 143 kDa. In order to unravel the enzymatic activity of the MDG and to elucidate the function associated with the different domains, the full length protein (GH5-12-12-CBM2-2, without signal peptide) along with the three C-terminal truncated versions of the MDG were cloned (via In-Fusion cloning) and expressed in *E. coli* using the pET expression system (pET24a, C-terminal His_6x_-tag). The truncated versions were: GH5-12-12 (missing the two CBM2s), GH5-12 (missing two CBM2s and the second GH12) and single GH5 (missing all other domains) (**Figure 1**).

All four proteins were recombinantly expressed in *E. coli* BL21 (DE3)-CodonPlus-pRIL. The truncated versions GH5 and GH5-12 were formed as fully soluble proteins, whereas only a small portion of the full length MDG (GH5-12-12-CBM2-2) and GH5-12-12 was obtained in the soluble protein fraction as demonstrated by SDS-PAGE and immunoblotting with His-tag specific antibodies (**Supplementary Figure S4**). Additional efforts to improve the soluble expression of the MDG and GH5-12-12 using different expression strains (e.g., *E. coli* Rosetta and *E. coli* SoluBL21) or expression conditions, e.g., lower temperature and different culture media, were not successful. Also purification from the membrane fraction using denaturation (e.g., urea, triton X-100) and renaturation approaches did not significantly improve the yield and purity of either protein. Due to the low yield of soluble protein, the MDG and GH5-12-12 were only partially purified and the enzymatic assays were performed with the soluble fraction after heat precipitation. From 8 g (wet weight) of recombinant *E. coli* cells, 12.4 mg and 9.6 mg of the MDG and the GH5-GH12-GH12 protein were obtained. For the soluble proteins GH5 and GH5-12, a purification protocol via heat precipitation (60°C, 30 min), ammonium sulfate fractionation [GH5, 2.2 M (NH_4_)_2_SO_4_; GH5-12, 2.6 M (NH_4_)_2_SO_4_], followed by ion exchange and size exclusion chromatography was established (**Supplementary Figure S5**). Notably, although all four proteins were cloned with a C-terminal His-tag, none of the proteins bound to Ni-TED columns. The purification procedure yielded 10.4 and 6.9 mg of essentially pure GH5 and GH5–GH12 protein, from 15 g (wet weight) of recombinant *E. coli* cells, respectively. For storage of protein, samples were supplemented with glycerol 20% (v/v) and frozen at -70°C.

### Cellulolytic Activities of the Full Length and Truncated MDG Versions

**FIGURE 3:**
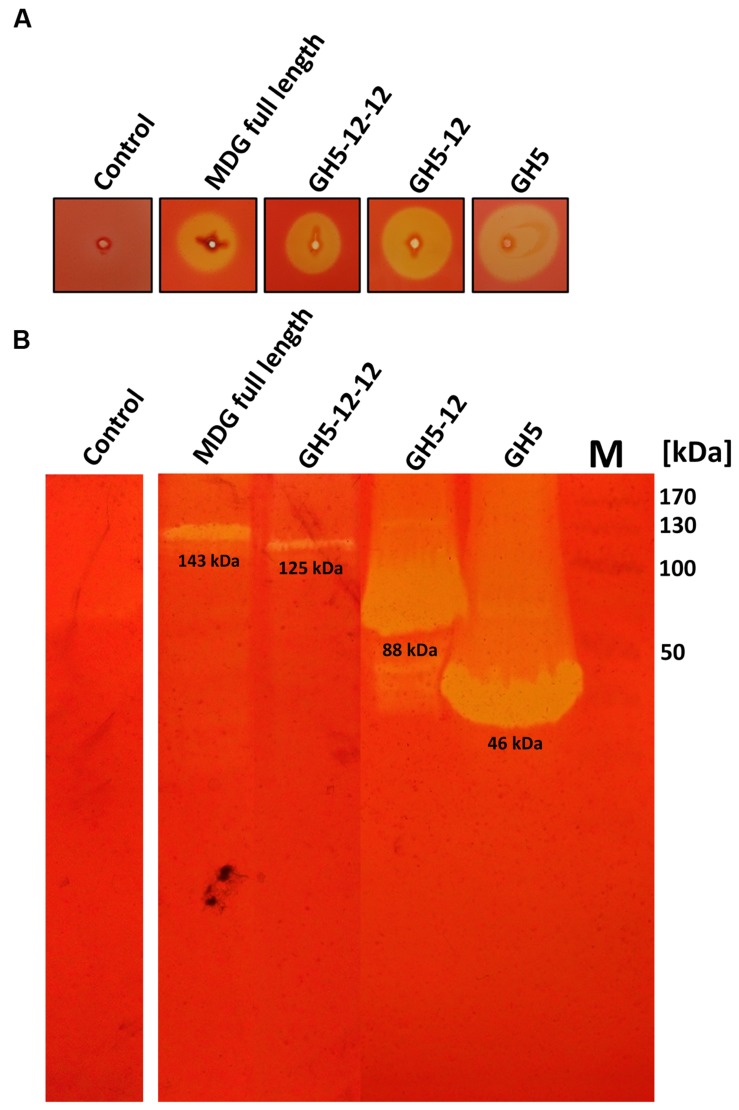
**Cellulolytic activities of full length and truncated MDG proteins.** Hydrolytic activities were analyzed on CMC-agar-plates **(A)** and in CMC-zymogram gels **(B)**. For CMC screening plates 10 μL (5–10 μg of total protein after heat precipitation at 60°C) and for zymogram gels 20 μL (3 μg of protein after heat precipitation at 60°C (GH5 and GH5-12) or 2 μg of solubilized membrane fraction (GH5-12-12 and MDG full length)) were applied. As negative control, crude extract of *E. coli* BL21 (DE3) pRIL with vector pET24a without insert was used. Marker, M **(B)**: prestained protein standard (Fermentas), GH5 (∼46 kDa) and GH5-12 (∼88 kDa), GH5-12-12 (∼125 kDa) and MDG full length (∼143 kDa).

For all four proteins, activity with 0.2% (w/v) CMC was detected on substrate agar plates and in zymogram gels (**Figure 3**). In the zymogram gels, the enzyme activity correlated to protein bands of the expected size for the full length and truncated MDG proteins (**Figure 3B**).

### Substrate Specificity of the Full Length and Truncated MDG Proteins

**FIGURE 4:**
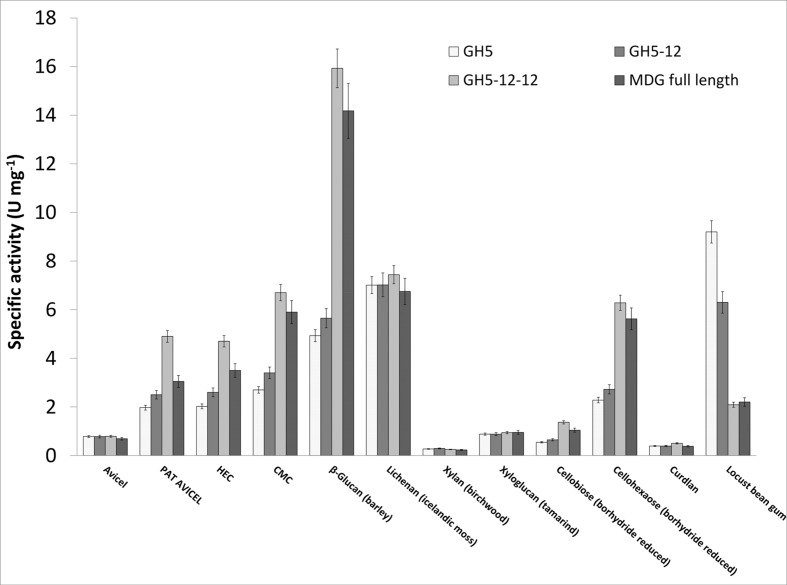
**Substrate specificity and specific activity of the full length MDG and truncated MDG proteins.** Enzyme activities were determined using the DNS assay. Substrate solutions [1% (w/v)] were incubated for 30 min with 33 μg of enriched protein fractions at 60°C. For GH5 and GH5-12 purified enzymes (after storage at -70°C) and for the full length MDG and GH5-12-12 enriched protein fractions after heat precipitation were used. microcrystalline cellulose (MCC) Avicel phosphoric acid treated (PAT) Avicel, hydroxyethyl cellulose (HEC), carboxymethyl cellulose (CMC).

The substrate specificity of the partially purified full length MDG and GH5-12-12 protein as well as the pure GH5-12 and GH5 versions was investigated using the DNS assay (Bernfeld, 1955) under standard conditions (60°C, pH 6.0) (**Figure 4**, **Supplementary Table S4**).

For all four proteins, the highest activity was observed on mixed β-1,3/1,4-glucans, i.e., barley β-glucan (4-16 U mg^-1^) and lichenan from icelandic moss (7 U mg^-1^). Endo-β-1,4-glucanase activity was also high on β-1,4-linked glucans such as CMC (3–7 U mg^-1^), hydroxyethyl cellulose (2–5 U mg^-1^), phosphoric acid treated (PAT) Avicel (2–5 U mg^-1^) and Avicel (1 U mg^-1^). In addition, all four proteins exhibited significant activity on cellooligosaccharides (cellohexaose (2–6 U mg^-1^) but only minor activity with cellobiose (0.5–1 U mg^-1^). TLC analysis of cellobiose and oligosaccharides (3–6 glucose units, G3 – G6) hydrolysis products revealed cellobiose as a main product for all four proteins. In addition, some glucose was detected for all proteins and cellotriose for full length MDG and GH5-12-12 (**Supplementary Figure S6**). Very low activity (<1 U mg^-1^) was observed for xylan (birchwood, β-1,4 linked D-xylose units), xyloglucan (β-1,4 linked D-glucose backbone with α-1,6 linked D-xylose side chains) and curdlan (β-1,3-glucan, 0.4 – 0.5 U mg^-1^). Notably high activity (2–9 U mg^-1^) was also observed on galactomannan, i.e., locust bean gum (LBG, β-1,4 linked D-mannose backbone with α-1,6 linked D-galactose side chains) suggesting mannan endo-1,4-β-mannosidase or β-mannosidase activity.

### Characterization of the Full Length MDG and the GH5 Protein

**FIGURE 5:**
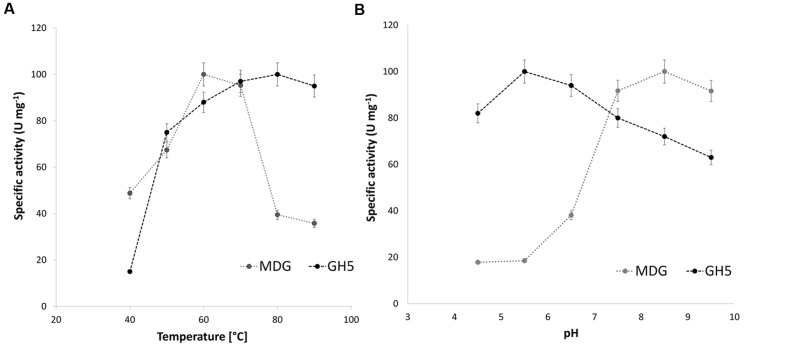
**Effect of temperature **(A)** and pH **(B)** on the specific activity of the full length MDG and GH5 protein.** Temperature and pH-dependent activity was determined with barley β-glucan [1% (w/v)] as substrate using the DNS assay. Substrate solutions were incubated for 30 min with 44 μg of enriched protein fractions after heat precipitation (MDG) or 28 μg of purified protein (GH5) at the indicated pH value (at 60°C) or temperature (at pH 7) in McIlvaine buffer (0.2 M Na_2_HPO_4_ titrated with 0.1 M citric acid).

For industrial applications the temperature and pH optima of enzymes as well as their stability toward detergents are of high interest. The respective enzymatic properties were analyzed for the partially purified full length MDG as well as the purified GH5 protein with barley β-glucan as substrate using the DNS assay. The enzymatic activities were determined in a temperature range from 40°C to 90°C and a pH range from 4.5 to 9.5 (**Figure 5**).

The full length MDG showed the highest activity (4 U mg^-1^) at a temperature of 60°C and possesses a pH-optimum from 7.5 to 9.5 with a maximum activity of 8 U mg^-1^ pH 8.5. In contrast the single GH5- protein showed the highest activity at 90°C and exhibited a broad pH optimum (pH 4.5–9.5) with highest activity at pH 5.5. Therefore, the truncation seems to broaden the temperature range to higher temperatures as well as the pH range to more acidic pH-values.

In order to establish a purification protocol, the MDG was solubilized from the *E. coli* membrane fraction by treatment with 8 M urea. Interestingly, the enzyme was active even after 2 days of incubation in the presence of 8 M urea (data not shown). These results indicate a high stability of the MDG toward denaturing agents.

## Discussion

Archaea of the genus *Thermococcus* are widespread in shallow water and deep-sea marine environments, as well as in deep-subsurface habitats. At present, this genus comprises more than 30 species with a G + C content ranging from 40.2 to 56.1%. Phenotypically *Thermococcus* species are very similar: they all are obligate anaerobes degrading peptides or, less frequently, polysaccharides, and use elemental sulfur as a terminal electron acceptor (Schut et al., 2014). Among *Thermococcales*, the ability to grow with C1 compounds (CO and formate) coupled with hydrogen production seems to be restricted to deep-sea representatives possessing corresponding enzyme clusters (Sokolova et al., 2009).

Strain 2319x1 was isolated from the hot vent, located in the tidal zone of Kunashir Island, Southern Kurils. Organic matter of plants, alga and animals is brought to the vent with tidal water providing a constant supply of substrates for hyperthermophilic microorganisms inhabiting sand and water of the vent. In addition, to proteinaceous substrates, which are common energy sources of all *Thermococcales*, strain 2319x1 degrades a wide spectrum of sugars and polysaccharides, including highly recalcitrant higher plant polysaccharides, like cellulose and xylan. These substrates are more abundant in terrestrial hot springs predominantly inhabited by organotrophic *Crenarchaeota*. Accordingly, the ability to degrade cellulose and xylan has been identified so far mainly in this archaeal phylum (**Supplementary Figure S1**).

*Thermococcus* sp. strain 2319x1 isolated from the organics-rich tidal zone is, to our knowledge, the first representative of the phylum *Euryarchaeota* and the first marine hyperthermophilic isolate capable to grow efficiently on cellulose or xylan. The analysis of cell-bound exoenzyme activities, of cells grown on different polysaccharides, compared to cells grown on gelatin revealed induction of exoenzyme expression in response to polysaccharides although the observed polysaccharolytic activity is not always clearly linked to the growth substrate (**Figure 2**). In its natural habitat, this exoenzyme repertoire will allow the strain 2319x1 to utilize allochthonous organic matter originating from plants or algae. The growth yield of AMC grown cells of strain 2319x1 is the highest among known hyperthermophilic cellulolytic archaea (**Supplementary Figure S7**).

### Genome-Scale Metabolic Reconstruction of the Central Carbohydrate Metabolism

*Thermococcus* sp. strain 2319x1 shares several metabolic features with well-characterized members of the order *Thermococcales*, i.e., *P. furiosus* and *T. kodakarensis* (Bräsen et al., 2014), but is unique in regard to its broad sugar substrate spectrum, especially in its capability to grow on xylan, xyloglucan, cellulose as well as on monosaccharides, such as D-glucose and D-xylose. The spectrum of sugars utilized by *P. furiosus* and *T. kodakarensis* is restricted to maltose (*P. furiosus* only) and to higher maltooligosaccharides, whereas glucose cannot be used. According to the broad substrate specificity and physiological versatility of *Thermococcus* sp. strain 2319x1, numerous genes encoding GHs, GEs and GTs of different enzyme families were identified in its genome (**Supplementary Table S3**). Due to its broad substrate specificity (**Table 1**), the presence of sugar transporters is extremely important for strain 2319x1. In *T. kodakarensis*, only one oligosaccharide ABC transporter has been shown to be essential for carbohydrate uptake (Matsumi et al., 2007), whereas in *P. furious* three ABC transporters for trehalose/maltose, maltodextrin and cellobiose have been characterized (Koning et al., 2001; Lee et al., 2003). In the genome of *Thermococcus* sp. strain 2319x1 a number of genes encoding ABC transporters were identified. For three of them, the transport of either D-glucose/D-xylose (ADU37_CDS01060-01090), cellobiose/cellooligosaccharides (ADU37_CDS07290-07330) or maltodextrins (ADU37_CDS18970-18930), respectively, is annotated based on sequence similarities to the characterized homologs (Xavier et al., 1996; Koning et al., 2001; Erbeznik et al., 2004). Notably, one of two SSS transporter genes found in the strain 2319x1 genome (ADU37_CDS10740), exhibits high similarity to the characterized human myoinositol:Na^+^ symporter SMIT2, which efficiently transports both D-glucose and D-xylose, and might therefore represent a glucose:Na^+^ symporter (2.A.21.3.2) (Coady et al., 2002).

Like in other anaerobic *Thermococcales*, D-glucose is degraded via a modified EMP pathway, and acetate, CO_2_ and H_2_ are the fermentation products (Siebers and Schönheit, 2005; Bräsen et al., 2014). Pentoses are formed from fructose-6-phosphate via the reversed ribulose monophosphate pathway. The formation is catalyzed by a fused D-arabino-3-hexulose-6-phosphate formaldehyde-lyase and phosphohexuloisomerase as described for *T. kodakaraensis*, and an incomplete non-oxidative pentose phosphate pathway (Kato et al., 2006; Orita et al., 2006; Bräsen et al., 2014). However, the central metabolic pathway for D-xylose degradation still remains to be elucidated. Homologs were neither found for the reported archaeal nor for the bacterial degradation pathways.

### Enzymatic Properties of the MDG of *Thermococcus* sp. Strain 2319x1

The analysis of hydrolases capable of degrading β-linked polysaccharides revealed two candidates in the genome sequence of strain 2319x1, i.e., the GT35 family representative ADU37_CDS05640 and ADU37_CDS22600. The latter is a MDG with so far unknown, unique domain architecture (GH5-12-12-CBM2-2), which has not been observed in any of the publicly available database entries. Homologs of the few known archaeal cellulases or xylanases could not be identified in strain 2319x1. Also, related polysaccharide hydrolyzing enzymes of other enzyme families were absent.

The GT35 enzyme, purified from *Themococcus zilligii*, was previously characterized as a xylanase (Uhl and Daniel, 1999). However, addition of xylan to the medium neither supported growth of *T. zilligii* nor enhanced its xylanase activity. In addition the enzyme sequence was highly similar to maltodextrin phosphorylases, e.g., from *T. litoralis*, raising questions about its enzymatic activity (Rolland et al., 2002). The GT35 sequence of strain 2319x1 (ADU37_CDS05640) is highly similar to the characterized maltodextrin phosphorylase from *T. litoralis* (96.7% identity) (Xavier et al., 1996). Less pronounced similarity was observed to the putative xylanase from *T. zilligii* (75.8% amino acid sequence identity), suggesting maltodextrin phosphorylase activity for this protein.

Thus, the only remaining predicted enzyme, capable of hydrolyzing β-linked polysaccharides was ADU37_CDS22600. In order to unravel the enzymatic function of the MDG as well as its different protein domains, the encoding gene as well as three truncated versions encoding proteins with reduced domain complexity were cloned and expressed in *E. coli*. The full length protein MDG as well as the GH5-12-12 protein were only partially soluble and despite many efforts, only a partial purification by heat precipitation was achieved (**Supplementary Figure S4**). For the soluble GH5-12 and GH5 proteins a successful purification protocol was established (**Supplementary Figure S5**). The activities of all recombinant proteins were confirmed by substrate agar plates, as well as by zymography with CMC as substrate (**Figure 3**).

All four proteins were highly active against the mixed β-1,3/1,4-glucans, barley β-glucan and lichenan. So far, only few archaeal endoglucanases have been reported to hydrolyze such substrates (**Supplementary Table S4**; **Figure 4**). The activity against curdlan was low, indicating a high specificity of all catalytic domains of ADU37_CDS22600 to β-1,4-glucosidic bonds. The activity against microcrystalline cellulose (MCC) Avicel, phosphoric acid treated (PAT) Avicel, HEC and especially CMC suggests that all four proteins act as endoglucanases (Béguin and Aubert, 1994). Furthermore, the specific activity increased with increasing domain complexity for all substrates containing more than one consecutive β-1,4-glycosidic bond. For substrates containing none or no more than one consecutive β-1,4-glycosidic bond (i.e., curdlan, xylan, galactomannan or lichenan, respectively), no effect was observed. Also α-1,6-linked side chained substrates (xyloglucan) or insoluble substrates (Avicel) were not converted more efficiently by the higher complexity variants MDG and GH5-12-12 of ADU37_CDS22600. This suggests that the addition of the second GH12 domain has a positive influence on enzyme activity and that this second GH12 domain probably acts as exoglucanase and β-glucosidase, enabling a more efficient hydrolysis of substrates. Hence, the full-length MDG seems to act in a processive mode. Since for both proteins MDG and GH5-12-12 only partially purified enzyme fractions were used, compared to the purified GH5 and GH5-12 proteins, the specific activity is even underestimated. However, we have no information if possible structural changes in the multidomain enzyme or additional enzyme activities (e.g., exoglucanase activity) provided by the second GH12 domain are responsible for the enhanced enzyme activity. Therefore, for a final evaluation, future studies with the two single GH12 domain enzymes have to be awaited.

In the presence of cellooligosaccharides (i.e., cellotriose to cellohexaose), the formation of cellobiose as the main product and of some glucose was shown for all four proteins. Cellotriose was identified as additional product only for the full length MDG and GH5-12-12. Notably, additional activity with galactomannan (LBG) was observed. In contrast to the increase in glucanase activity with increasing domain complexity, the additional β-(endo)-mannosidase activity seems to be linked to GH5 domain. Hence, the combination of different enzyme domains in MDG extends the substrate specificity of the enzyme and seems to allow the processive conversion of various polysaccharides differing in size and structure.

The substrate specificity of the MDG protein was rather unexpected, since *Thermococcus* sp. strain 2319x1 showed no growth on barley β-glucan as well as on galactomannan, and the enrichment was performed on xylan, which is, if at all, a minor substrate of the enzyme. However, significant growth was observed on CMC and AMC suggesting that the enzyme has a major role in cellulose degradation. This assumption is in agreement with determined exoenzyme activities with β-glucan, AMC, CMC, xylan and xyloglucan as substrates of xylan-, AMC- and xyoglucan-grown cells.

The detailed characterization of the full length MDG revealed a slightly alkaline pH optimum (7.5–9.5) and a rather low temperature optimum (60–70°C), compared to the optimal growth temperature and pH of the organism (75–85°C, pH 7.0). In contrast, the single GH5 protein exhibits a slightly acidic, broad pH optimum (4.5–9.5) and high temperature (90°C) optimum, consistent with the respective growth requirements of the organism. However, the expression of the MDG in *E. coli* is challenging and only a very small fraction of the MDG and GH5-12-12 protein was soluble, probably due to the size of the protein and the complex domain structure. Also, for several CBMs, the need of glycosylation has been reported (Boraston et al., 2003; Chen et al., 2014) and the archaeal system generating these kinds of modification is absent in the bacterial host. Preliminary studies on the binding to mircrocrystalline cellulose (MCC) Avicel of the full length recombinant MDG compared to its single GH5 domain revealed no obvious difference in binding affinity (data not shown). Therefore, the different optima of MDG and GH5, the different solubility, and the similar binding patterns to MCC (unexpected because of the presence and absence of the CBM domains in MDG and GH5, respectively) might be caused by the heterologous expression in *E. coli* which does not allow for the required posttranslational modifications.

### Phylogenetic and Functional Implications for the Single MDG Domains

The presence of the MDG with an unusual domain complexity raises questions about the evolutionary origin and the relationship of the different protein domains to available functionally characterized proteins. BLASTp searches revealed the highest identity of the GH5 domain (position 56–385) to the characterized endo-1,4-β-glucanase from the close relative *P. horikoshii* (PH1171, EGPh, 86% identity). However, in contrast to the artificial GH5 domain from the strain 2319x1 MDG, the *P. horikoshii* enzyme possesses the highest activity with CMC (100%), but much lower activity with lichenan (43.9%) and very low activity with β-glucose oligomers (cellobiose to cellopentaose, <2%) (Ando et al., 2002; Kashima et al., 2005). PH1171 showed a pH optimum between 5.4 and 6.0, a temperature optimum at 97°C, and a specific activity of 8 U mg^-1^ [0.5% (w/v) CMC, pH 5.6, 85°C] (Ando et al., 2002). For PH1171 processive hydrolytic activity with direct formation of cellobiose by random cleavage was reported (Kim and Ishikawa, 2010). Also, structural prediction tools (HHpred^4^; Söding et al., 2005) revealed the highest structural similarity of the GH5 domain to the crystal structure of PH1171, which exhibits a typical triosephosphate isomerase (TIM) (αβ)_8_ barrel fold (Kim and Ishikawa, 2010, 2011). The eight residues found in the catalytic cleft of PH1171 as well as in other family 5 enzymes (*Pho*: Arg102, His155, Asn200, Glu201, His297, Tyr299, Glu342 and Trp377) as well as the four cysteines (*Pho*: Cys106-Cys159, Cys372-Cys412) involved in disulfide bond formation, are conserved in the GH5 domain (Kim and Ishikawa, 2010, 2011) (**Supplementary Figure S8**).

The second GH12-2 domain, comprising the amino acid residues 902–1070 of the full length MDG, is highly similar to the well characterized endo-1,4-β-glucanase encoded by ORF PF0854 from *P. furiosus* (EGPf, 85% idenitity). Although this ORF appears also as closest homolog of the first GH12-1 domain (position 580–756 of full length MDG) in BLAST searches, the amino acid identity is much lower (34% identity). Like the MDG, PF0854 cleaves β-1,4-bonds in mixed linkage glucans (β-1,3/1,4-bonds) and in cellulose (β-1,4-bonds). Barley-glucan, lichenan, and to a lesser extend CMC and Avicel PH101 have been reported as substrates (Bauer et al., 1999). However, the highest specific activity has been reported toward β-1,4-linked cellulose oligosaccharides, i.e., cellotetraose, cellopentaose and cellohexaose. Additionally, a function in attacking the β-1,4-amorphous insoluble regions within the insoluble cellulose was suggested (Bauer et al., 1999).

Moreover, two characterized GH12 enzymes from the hyperthermophilic bacterium *Thermotoga maritima* were also among the most similar characterized homologs. *Thermotoga maritima* enzymes were characterized as intracellular endo-β-1,4-glucanase TM1524 and extracellular exo-β-1,4-glucanase TM1525. Both enzymes exhibit similar substrate specificity on a variety of β-linked biopolymers (CMC, β-glucan, and *p*-nitrophenyl-β-D-cellobioside) (Bronnenmeier et al., 1995; Liebl et al., 1996; Vanfossen et al., 2008), but differ in their pH optima, long-term thermostability, as well as in stability in the presence of salts (NaCl). Both genes are in juxtaposition on the genome and a recent gene duplication event has been proposed (Liebl et al., 1996). The crystal structure of TM1524/Cel12 from *T. maritima* was solved (Cheng et al., 2011).

Another characterized homolog is the GH12 xylanase/cellulase from S. solfataricus (SSO1354), which exhibits similar activity on various xylans, CMC and arabinan (Maurelli et al., 2008). Although this protein was not active against Avicel, mannan and xyloglucan, its activity spectrum indicates the possibility of a single GH12 domain being active against various polysaccharides including xylan that differ in size and structure.

HHpred analyses (Söding et al., 2005) revealed that both, the GH12-1 and the GH12-2 domain, of the 2319x1 full length MDG show a high structural similarity to the solved PF0854 structure, which adopts a compact β-jellyroll fold with a calcium-binding motif (DxDxDG) (Kim et al., 2012). The catalytic residues, the nucleophile PF0854-E197 and the proton donor PF0854-E290, conserved in family 12 GHs, are found in both MDG-GH12 domains. In contrast the calcium-binding motif, important for activity, thermostability and protein folding, as well as the residues involved in metal ion coordination (PF0854: Asp68, Asp70, Asp72, Asn74, Glu76, Asp142), are only present in GH12-2 domain. Notably the metal ion coordinating residues, except Asp142, were found in the linker region in front of the GH12-2 domain, which shows only low sequence similarity (**Supplementary Figure S9**).

Carbohydrate-binding modules enable enzymes to attach to polymer surfaces and, thus, to increase the local substrate concentration and to enhance the catalytic efficiency (Simpson et al., 2000). CBMs are not common for archaeal glycosidases. Among all 1953 CBM2 accessions currently available in the CAZy database, only six are from Archaea. Both the *P. horikoshii* GH5 and the *P. furiosus* GH12, do not possess cellulose binding domains. The CBM2-1, comprising residues 1103–1199 of the full length MDG, shows the highest similarity (BLASTP) to the CBM of the endo-1,4-β-endoglucanase Cel12E from an unidentified prokaryotic organism (37% identity) and to the CBM of the chitinase PF1233 from *P. furiosus* (35% identity) (**Supplementary Table S5**). Cel12E has recently been reported to be of archaeal origin and also exhibits an unusual though less complex N- to C-terminal GH12-CBM2-CBM2 domain architecture. All three domains of Cel12E show significant similarity to the thermococcal homologous (Leis et al., 2015). However, the CBM2-2, i.e., residues 1208–1303 of the full length MDG, shows statistically reliable hits only to bacterial proteins with the highest similarity to the CBM of the chitinase from *Mycobacterium kansasii* (35% identity).

CBM2 represents one of the largest CBM families in CAZy and comprises members that can bind to cellulose, xylan and chitin. CBM2 domains are β-sandwich fold domains, typically comprised of two four stranded sheets that contain a planar face that interacts with a ligand via a hydrophobic strip of aromatic residues (Xu et al., 1995). Structural searches (HHpred) predict 25% (CBM2-1) and 26% (CBM2-2) identity to the cellulose-binding domain of the exo-β-1,4-glucanase from *Cellulomonas fimi* (PDB 1exg_A), and 29% (CBM2-1) and 19% (CBM2-2) identity to the chitinase-binding domain of the *P. furiosus* chitinase (Xu et al., 1995; Simpson et al., 2000; Nakamura et al., 2008) (**Supplementary Figure S10**).

Additional phylogenetic implications come from genomic context analysis in *Thermococcus* sp. strain 2319x1. The *mdg* gene is localized in the genome together with three other genes, encoding one putative GT ADU37_CDS22610 and two hypothetical proteins, which are flanked by two transposases encoding genes (ADU37_CDS22570-22590 and ADU37_CDS22640-22650) (**Supplementary Figure S11**), supporting lateral gene transfer to the strain 2319x1 or its closest ancestor. Moreover, as outlined above, individual domains of the full length MDG (ADU37_CDS22600) have different sets of nearest relatives (BLAST hits), indicating their different origin. While the GH5 and the GH12-2 domains have highly similar homologs, the GH12-1 and the CBM2-2 domains have less similar homologs, all from the bacterial domain. The CBM2-1 domain has less than ten statistically significant hits, mainly from thermococcal/pyrococcal chitinases (**Supplementary Table S5**). Phylogenetic analysis of the individual MDG glycosidase domains thus supports their different derivation (**Supplementary Figures S12** and **S13**). The GH5 domain was, most probably, horizontally transferred from the ancestors of *Xanthomonadales*, and the GH12-2 domain from *Thermotogales*. The GH12-1 domain of strain 2319x1 MDG was possibly also acquired from *Thermotogales*, but presumably much earlier as indicated by the reduced similarity. Our findings are in accordance with the general view on evolution of GHs-encoding genes in *Archaea*, that regards horizontal gene transfer as the determining factor behind archaeal GH repertoires (Henrissat et al., 2009).

In general, the identification of new thermostable cellulases with appropriate performance and/or novel functionalities offers great potential for biotechnological applications. Thus for the endo-1,4-β-glucanase from *P. horikoshii* (PH1171, GH5 family) in combination with β-glucosidase from *P. furiosus* (PF0073, Bauer et al., 1996), a complete saccharification of cellulose [phosphoric acid treated (PTA) Avicel] to glucose (incubation for 3 days at 85°C) was demonstrated (Kim and Ishikawa, 2010). In contrast the endo-1,4-β-glucanase from *P. furiosus* (PF0854, GH12 family, forming G2, G3, and G4 oligosaccharides) and PF0073, from the same organism, allowed for complete saccharification of barley glucan and lichenan to glucose (Kataoka and Ishikawa, 2014).

## Conclusion

Here we describe a novel multidisciplinary approach for the screening of hydrolases of biotechnological interest, based on *in situ* enrichments using selected substrates, (comparative) genomics and biochemical characterization of enzymes. *Thermococcus* sp. strain 2319x1 was isolated from the tidal hydrothermal spring, rich in organic matter. It is the first archaeon growing on xyloglucan, one of few hyperthermophilic archaea growing on cellulose and xylan, and the first euryarchaeon growing on xylan. While the D-glucose utilization pathway by strain 2319x1 seems to proceed as described for other *Thermococcales*, the D-xylose utilization pathway probably involves novel steps and requires further experimental analysis. Genomic analysis revealed a single gene, annotated as a glycosidase, capable of degrading β-glycosides. This protein (MDG) possesses a unique multidomain organization and acts mainly as endoglucanase and β-(endo)-mannosidase with various (although very low) side activities including xyloglucanase, xylanase, 1,3-glucanase. The GH5 domain of MDG exhibits mainly endoglucanase and β-(endo)-mannosidase activities, while the GH12 domains presumably give additional multifunctionality to the MDG. The two CBM2 domains of this enzyme probably allow its attachment and binding to insoluble polysaccharides. Altogether, this domain organization seems to allow a processive hydrolysis of various polysaccharides, including insoluble ones. The alkaline pH optimum (pH 8.5) of the MDG, as well as its multifunctionalty, might be of interest for future biotechnological applications, since most reported thermostable endoglucanases possess more acidic pH range (**Table 2**). Horizontal gene transfer is regarded as the determining factor behind archaeal GH repertoires (Henrissat et al., 2009). The genomic context of the MDG-encoding gene supports its proneness to horizontal gene transfer. Moreover, phylogenetic analysis of the individual protein domains of the MDG approved that the unusual domain architecture was gained by independent HGT events (“tinkering”) from different bacterial phyla, i.e., *Proteobacteria*, *Actinobacteria*, and *Thermotogae*. Massive horizontal gene transfer from *Thermotogales* to *Thermococcales* and, more general, from Bacteria to Archaea has been reported previously (Nelson et al., 1999; Nelson-Sathi et al., 2015).

**Table 2:** Characterized endoglucanases and endoxylanases from hyperthermophilic Archaea.

Microorganism	Characterized enzyme	Uniprot accession	Domain organization	T_opt_°C	pH	Substrates	Reference
*Thermococcus* sp. strain 2319õ1	Multifunctional glycosidase		GH5-12-12-CBM2-2	60 (in *E. coli*)	8.5	CMC, amorphous cellulose, CE-cellulose, β-glucan, lichenan, xyloglucan, Avicel, xylan (beech, birch), locust bean gum, cellooligosaccharides (cellotriose to cellohexaose), cellobiose	This work


							
							
							
				90 (single GH5)	5.5 (single GH5)		
Enrichment culture (*Ignisphaera*)	Endoglucanase	F6M085	GH new GH-A clan + possibly 3 unknown domains	109	5.5	Lichenan, Avicel, CMC, β-glucan	Graham et al., 2011
*Sulfolobus solfataricus* MT 4 (DSM 5833)	Endoglucanase/xylanase	Q97YG7	GH12	90	7.0	xylan (oat, beech, birch), CMC, arabinan	Cannio et al., 2004 Maurelli et al., 2008
*Pyrococcus furiosus* (DSM 3638)	Endoglucanase	Q9V2T0	GH12	100	6.0	Cellotriose, cellopentaose, cellohexaose, β-glucan, lichanan, CMC	Bauer et al., 1999
*Pyrococcus furiosus* (DSM 3638)	Endo-1,3-β-glucanase	O73951	GH16	100–105	5.5–6.5	β-glucan, laminarin, lichenan	Gueguen et al., 1997
*Pyrococcus horikoshii* OT3	Endoglucanase	O58925	GH5	>97	5.4–6.0	CMC, Avicel, lichenan	Ando et al., 2002
*Thermococcus zilligi* AN1 (DSM 2770)	Xylanase^∗^	Q977U0	GT35	85	6.0	Xylan (larch, birch, oat), arabinoxylan (wheat)	Uhl and Daniel, 1999; Rolland et al., 2002
Environmental (*Thermococcus*)	Endolgucanase	A0A0F7YYA5	GH12 + CBM2 + CBM2	92	5.5	CMC, β-glucan, lichenan, AMC	Leis et al., 2015


*^∗^Questionable, see “Discussion” for details.*

## Author Contributions

SG, AS, TS, KZ, IK contributed to isolation, growth experiments, extracellular enzyme measurements of the new *Thermococcus* strain. PM, IK, and XP contributed to genome sequenzing and annotation of the genome sequence. CS, KJ, VK, CB contributed to cloning and enzymatic characterization of the multidomain cellulase. EB-O, BS designed the work. CS, IK, EB-O, and BS wrote the manuscript.

## Conflict of Interest Statement

The authors declare that the research was conducted in the absence of any commercial or financial relationships that could be construed as a potential conflict of interest.

## Footnotes

1 http://www.cazy.org/

2 http://www.genomics.cn/

3 http://www.cbs.dtu.dk/services/SignalP/

4 http://toolkit.tuebingen.mpg.de/hhpred
