# Supplementary material for: Isolation and Characterization of the First Xylanolytic Hyperthermophilic Euryarchaeon Thermococcus sp. Strain 2319x1 and Its Unusual Multidomain Glycosidase
Source: Front Microbiol. 2016 May 3;7:552. doi: 10.3389/fmicb.2016.00552 (PMC4853606; doi:10.3389/fmicb.2016.00552)
Supplement: Supplementary file 1 [file Data_Sheet_1.PDF]

Isolation and characterization of the first xylanolytic hyperthermophilic euryarchaeon *Thermococcus* sp. 2319x1 and its unusual multidomain glycosidase

Sergey N. Gavrilov[1#], Christina Stracke[2#], Kenneth Jensen[3], Peter Menzel[4], Verena Kallnik[2], Alexei Slesarev[1,5], Tatyana Sokolova[1],Kseniya Zayulina[1], Christopher Bräsen[2], Elizaveta A. Bonch-Osmolovskaya[1], Xu Peng[4], Ilya V. Kublanov[1]*, Bettina Siebers[2]*

[#]Both authors contributed equally
*Corresponding authors

[1]Winogradsky Institute of Microbiology, Research Center of Biotechnology, Russian Academy of Sciences, Moscow, Russia
[2]Molecular Enzyme Technology and Biochemistry (MEB), Biofilm Centre, Centre for Water and Environmental Research (CWE), University Duisburg-Essen, Essen, Germany
[3]Novozymes A/S, Bagsværd, Denmark
[4]Department of Biology, University of Copenhagen, Copenhagen, Denmark
[5]Fidelity Systems, Inc., Gaithersburg, MD USA

1. Introduction

Supplementary Table 1. Characterized and predicted cellulases and xylanases from hyperthermophlic Archaea.

Family	GH3	GH5	GH9	GH10	GH11	GH12	GH16	GH26	GH43	GH51	GH94
Activities	beta-xylo-gluco	beta-many (mainly endo)	beta-xylo-gluco (mainly endo)	endo-xylo	endo-xylo	beta-xylo-gluco (endo)	beta-many (mainly endo)	beta-many	xylo-arabino mainly	beta-xylo-gluco-arabino	beta-gluco-chito phosphorylases
# proteins from cultivated thermophilic archaea	**83**	**55**	**4**	**14**	**5**	**62**	**18**	**5**	**14**	**18**	**8**
**Crenarchaeota**											
Acidianus hospitalis W1						**1**					
Acidilobus saccharovorans 345-15	**1**	**1**				**3**					
Caldivirga maquilingensis IC-167	**2**	**1**				**5**					
Ignisphaera aggregans DSM 17230	**3**	**3**				**5**				**1**	**1**
Metallosphaera cuprina Ar-4						**1**					
Sulfolobus acidocaldarius (all strains)						**4**					
Sulfolobus islandicus (all strains)	**10**	**10**				**20**					
Sulfolobus solfataricus (all strains)	**5**	**5**				**12**					
Thermofilum carboxyditrophus 1505	**1**										
Thermofilum pendens Hrk 5	**1**	**1**				**1**					
Thermofilum sp. 1807-2	**1**										
Thermofilum sp. 1910b	**1**										
Thermoproteus uzoniensis 768-20		**1**				**2**					
Thermoproteus tenax Kra 1						**1**					
Thermosphaera aggregans DSM 11486							**1**				
Vulcanisaeta distributa DSM 14429		**1**				**1**					

**Euryarchaeota**							
Palaeococcus pacificus DY20341						**1**	
Picrophilus torridus DSM 9790	**1**	**1**					
Pyrococcus abyssi GE5		**1**					
Pyrococcus furiosus COM1						**1**	**1**
Pyrococcus furiosus DSM 3638						**1**	**1**
Pyrococcus horikoshii OT3		**1**					
Pyrococcus sp. ST04		**1**					
Thermococcus sp. ES1		**1**					
Thermococcus sp. AM4						**1**	
Thermococcus guaymasensis DSM 11113							**1**
Thermococcus sibiricus MM 739						**3***	**1**
Thermoplasma volcanium GSS1		**1**					

According to CAZy.

***** - very distant, not in CAZy

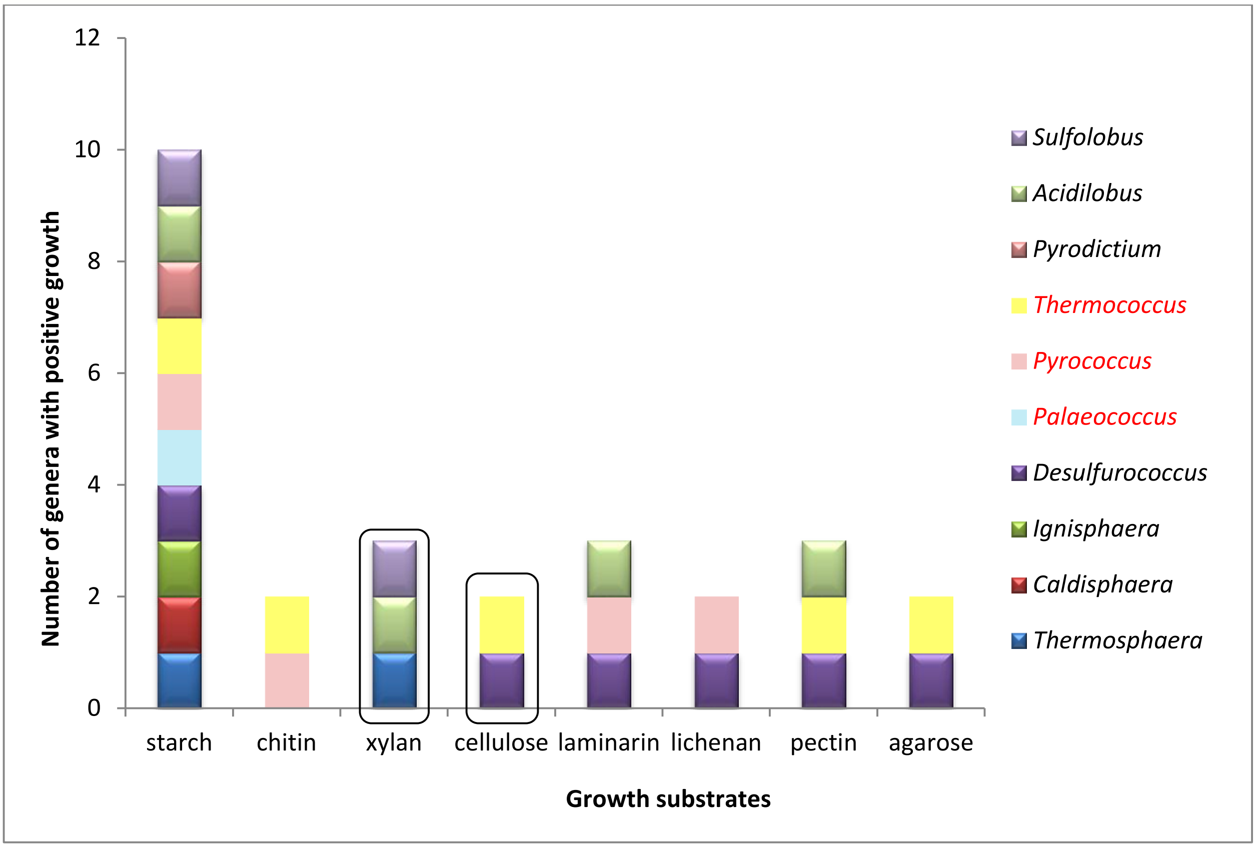

Supplementary Figure 1. Polysaccharide-degrading hyperthermophilic Archaea. Euryarchaeal genera are in red and their elements are in 2D; Crenarchaeal genera are in black and their elements are in 3D. Genera, growing on xylan or cellulose are boxed. Alpha-linked starch and its derivatives are widely utilized by hyperthermophilic archaea while beta-linked polysaccharides, such as cellulose and xylan are used rarely, mainly by representatives of the Crenarchaeota.

2. Material and Methods

2.1 Cloning, expression and purification of the MDG and truncated versions

Supplementary Table 2. Primers for In-Fusion® cloning of the *mdg* gene and its truncated versions. The 15 bp extensions complementary to the linearized expression vector pET24a are underlined.

Primer ID:	Sequence:
fwd-*mdg* (without signalpeptide)	5’-TCGCGGATCCGAATTATGACGACTGACACAAGCACG-3
rvs- *mdg* (GH5-12-12-CBM2-2)	5’-GTGCTCGAGTGCGGCAAGGATTTCGACTCCAAGGAG-3’
rvs- *mdg-trunc1* (GH5-12-12)	5’-GGTGCTCGAGTGCGGGAGCGAGACGTTGTAGAACC-3’
rvs- *mdg-trunc2* (GH5-12)	5’-GGTGCTCGAGTGCGGCTGGACCTCTCCGATGAGAAC-3’
rvs- *mdg-trunc3* (GH5)	5’-GGTGCTCGAGTGCGGCGGGGCAATGGCGTTTCCAG-3’

2.2 Purification of the GH5 and GH5-12 protein

Since an affinity chromatography by Ni-TED column revealed no binding of the His-tagged GH5 and GH5-12 proteins an alternative purification protocol via fractionated ammonium sulfate (AS) precipitation, ion exchange chromatography and size exclusion chromatography was established. For fractionated AS precipitation the protein fraction after heat precipitation was first incubated (30 min, stirring at 4°C) in the presence of 1 M AS and after centrifugation (26000 x g for 30 min at 4°C) the GH5 protein was precipitated with 2.2 M AS and the GH5-12 protein by addition of 2.6 M AS followed by centrifugation (26000 x g for 30 min at 4°C). After resuspension and dialysis against 20 mM TRIS HCl, $pH^{25^\circ C}$ 7.0 the protein fractions were applied on a Resource-Q column (6 mL column volume, flow rate 2 mL min^{-1}, GE Healthcare) equilibrated in 20 mM TRIS HCl $pH^{25^\circ C}$ 7.0, followed by washing with equilibration buffer (20-fold column volume) and elution with a linear salt gradient (0-1 M NaCl, 20-fold column volume, flow rate 2 mL min^{-1}, Äkta purifier, GE Healthcare). Fractions containing the respective proteins (checked by SDS-PAGE and activity tests) were pooled and concentrated via Vivaspin concentrators (30 kDa MW cut off, Sartorius Stedim Biotech). After dialysis overnight against 50 mM TRIS HCl, 300 mM NaCl, $pH^{25^\circ C}$ 7, both proteins were purified via size exclusion chromatography (Superdex™ 200, HiLoad™ 26/60 column, 320 mL volume, GE Healthcare) equilibrated in dialysis buffer (flow rate 2 mL min^{-1}, Äkta purifier, GE Healthcare). Protein fractions containing the purified protein (checked by SDS-PAGE and activity tests) were pooled and concentrated via Vivaspin concentrators (30 kDa MW cut off, Sartorius Stedim Biotech). The protein solutions were stored at -70°C (in the presence of 20% glycerol) and remained active for at least 5 month.

3. Results

Supplementary Table 3. Glycoside hydrolases **(**GHs) and carbohydrate esterases (CEs) encoding sequences, identified in the genome of *Thermococcus* strain 2319x1.

**№**	**GenBank ID**	**Cazy family**	**Pred Hel=**	**Topol ogy=**	**SignalP (G(+) & eukar)**
**1.**	ADU37_CDS00170	Glycoside hydrolase 13 family enzyme, alpha-amylase	0		N
**2.**	ADU37_CDS02890	Glycoside hydrolase 63 family enzyme, glycogen debranching enzyme, putative	0		N
**3.**	ADU37_CDS02900	Glycoside hydrolase 130 family enzyme, beta-1,4 mannooligosaccharide phosphorilase	0		N
**4.**	ADU37_CDS03850	Glycoside hydrolase 57 family enzyme, alpha-amylase/4alpha-gluconotranspherase, putative	0		N
**5.**	ADU37_CDS05320	Glycoside hydrolase 1 family enzyme, beta-glucosidase, putative	0		N
**6.**	ADU37_CDS05340	Glycoside hydrolase 57 family enzyme	0		N
**7.**	ADU37_CDS07270	Glycoside hydrolase 35 family enzyme,exo-beta D-glucosaminidase, putative	0		N
**8.**	ADU37_CDS07340	Glycoside hydrolase 1 family enzyme, beta-galactosidase, putative	0		N
**9.**	ADU37_CDS07440	Glycoside hydrolase 1 family enzyme, beta-galactosidase, putative	0		N
**10.**	ADU37_CDS07750	Glycoside hydrolase 122 family enzyme, alpha-glucosidase, putative	0		N
**11.**	ADU37_CDS12970	Glycoside hydrolase 57 family enzyme, 1,4alpha-branching enzyme	0		N
**12.**	ADU37_CDS13420	Carbohydrate esterase, putative	0		Y
**13.**	ADU37_CDS18940	Glycoside hydrolase 57 family enzyme, amylopullulanase, putative	1	o1075-1097i	Y
**14.**	ADU37_CDS18980	Glycoside hydrolase 13 family enzyme, neopullulanase, putative	0		N
**15.**	ADU37_CDS20530	Glycoside hydrolase 13 family enzyme	0		N
**16.**	ADU37_CDS21030	Glycoside hydrolase 13 family enzyme	1	i7-25o	Y
**17.**	ADU37_CDS21840	Putative oxidoreductase, related to GH109 family	0		N
**18.**	ADU37_CDS22600	Multidomain glycoside hydrolase protein, endoglucanase/endoxylanase	1	i7-29o	Y

PredHel and Topology= are results of transmembrane regions predictions by the TMHMM server (http://www.cbs.dtu.dk/services/TMHMM). "iXXX-oYYY" indicates the localization and coordinates of the transmembrane (TM) region in the protein with „i“ inside and “o” outside. TM predicitions at the N-terminal end of the protein often indicate the presence of a signal peptide (i.e. i7-25o in ADU37_CDS21030, i7-29o in ADU37_CDS22600). For ADU37_CDS18940 a C-terminal TM is predicted (protein region o1075-1097i).
SignalP (G(+) & eukar): results of signal peptide predictions by the SignalP server (for Gram-positives and Eukaryotes; http://www.cbs.dtu.dk/services/SignalP/index.php).

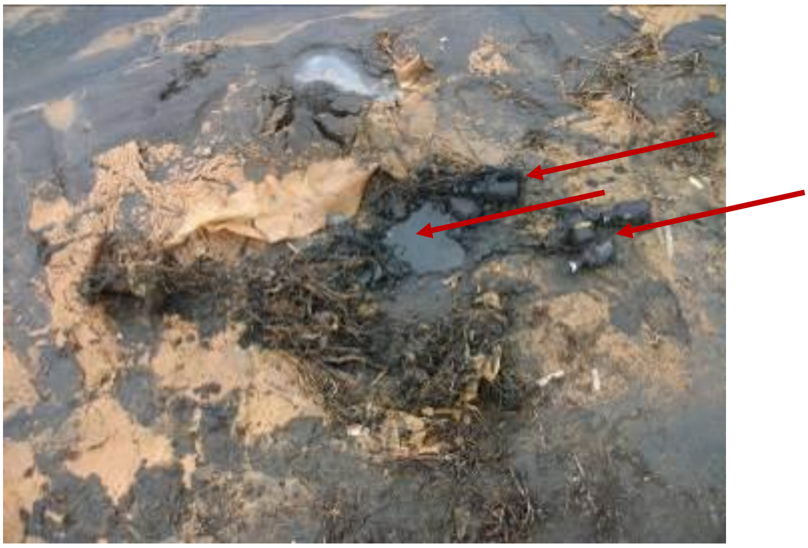

Supplementary Figure 2. Sampling site and *in situ* enrichment cultures. Arrows indicate the 50 mL bottles and 18 mL Hungate tubes incubated in the hot spring, located in the tidal zone near Goryachiy cape of Kunashir Island (South Kurils, Russian Far East region).

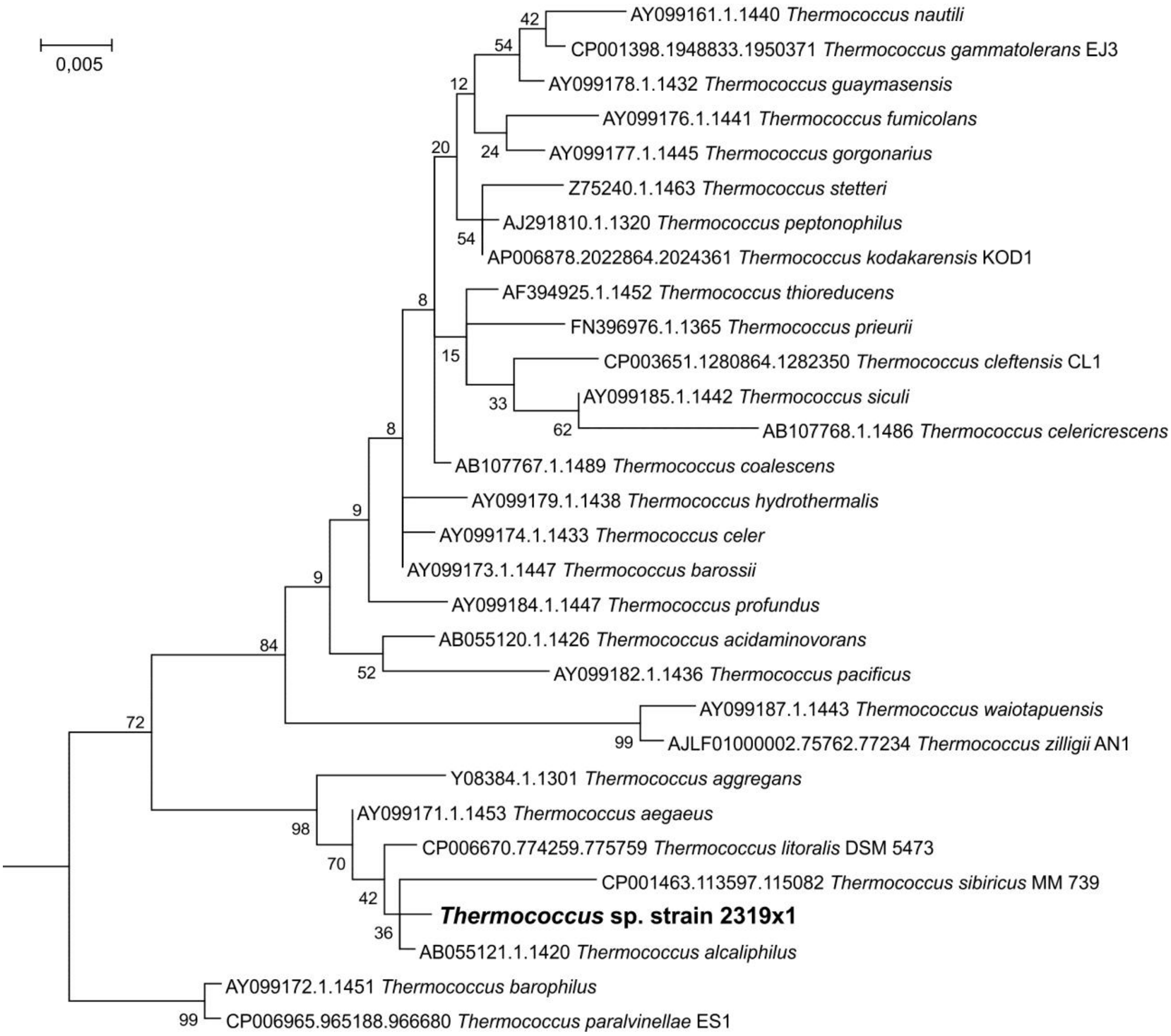

Supplementary Figure 3. 16S rRNA gene sequence-based Maximum likelihood phylogenetic tree of *Thermococcus* sp. strain 2319x1 and validly published representatives of the *Thermococcus* genus. The tree with the highest log likelihood (-3041.4022) is shown. The number of trees of totally 100 repetitions (bootstrap analysis) in which the associated taxa clustered together is shown next to the branches. The tree is drawn to scale, with branch lengths measured in the number of substitutions per site. The analysis involved 31 nucleotide sequences. All positions with less than 95% site coverage were eliminated. There were a total of 1286 positions in the final dataset. Evolutionary analyses were conducted in MEGA6 (Tamura et al., 2013). Bar, 0.50 nucleotides substitutions per 100. *Thermococcus* sp. strain 2319x1 is shown in bold. *Pyrococcus yayanosii* strain CH1 (NR 102853.1) was used as outgroup.

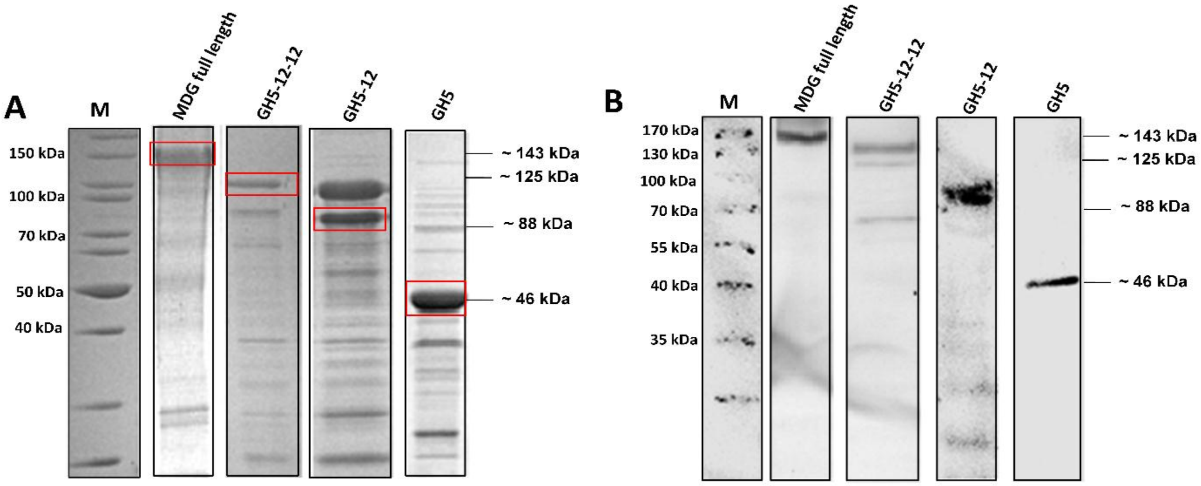

Supplementary Figure 4. Expression of the recombinant MDG and the different truncated versions in *Escherichia coli*. (A) Protein fractions (5 µg protein each lane) after heat precipitation at 60°C (30 min) were separated via SDS-PAGE (12.5% SDS-PAGE, coomassie staining) and (B) expressed proteins were identified by immunodetection via anti His-tag antibodies. Marker (M): unstained (A) and prestained (B) protein standard (Fermentas); MDG full length (~ 143 kDa); GH5-12-12 (~ 125 kDa); GH5-12 (~ 88 kDa); single GH5 (~ 46 kDa))

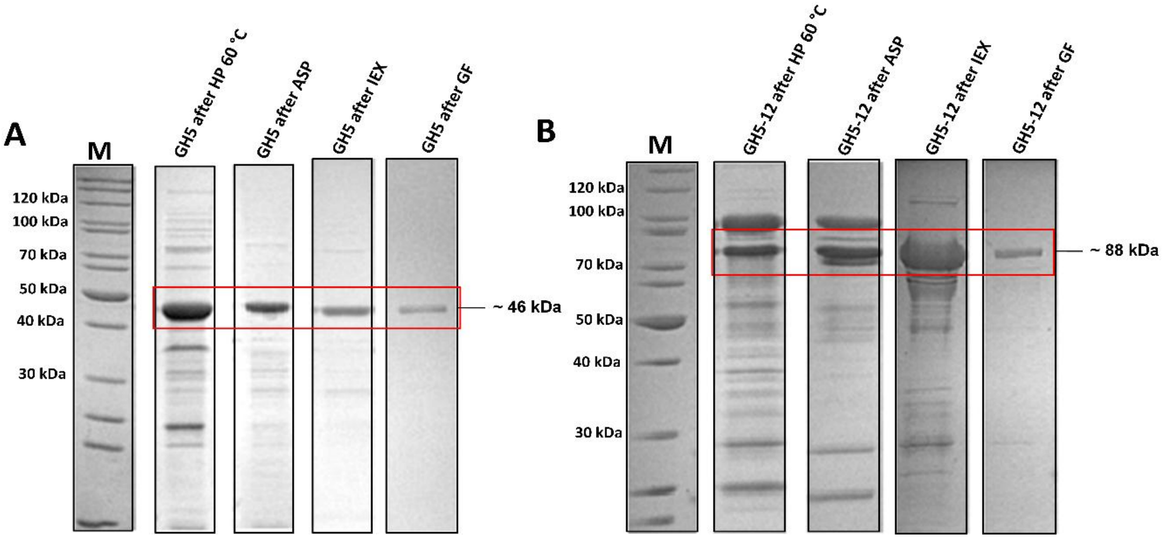

Supplementary Figure 5. Purification of the truncated GH5 (A) and GH5-12 (B) protein. Purification was followed by SDS-PAGE (12.5% SDS-PAGE, coomassie staining). Protein fractions (5 µg protein) after heat precipitation (HP), ammonium sulphate precipitation (ASP), ion exchange chromatography (IEX) and protein fractions (2 µg protein) after size exclusion chromatography (SEC) are shown. Marker (M) :unstained protein standard (Fermentas).

Supplementary Table 4. Substrate specificity and specific activity of the full length MDG and the truncated MDG proteins.

Substrate	Backbone linkage(s)	Specific activities [U mg^{-1}] (K_{cat} [s^{-1}])			
		GH5	GH5-12	GH5-12-12	MDG full length
**Glucan oligosaccharides**					
Cellobiose	β-1,4 only	0.5 (1498)	0.6 (3403)	1.4 (10248)	1.0 (8937)
Cellohexaose	β-1,4 only	2.3 (6293)	2.7 (14380)	6.3 (47120)	5.6 (48250)
**Glucan polysaccharides**					
Barley glucan	β-1,3/4	4.3 (13606)	5.6 (29832)	15.9 (119475)	14.2 (121664)
Lichenan	β-1,3/4	7.01 (19347)	7.02 (37065)	7.44 (39283)	6.75 (57915)
Carboxymethyl cellulose	β-1,4 only	2.7 (7452)	3.4 (17952)	6.7 (50250)	5.9 (50622)
Hydroxyethyl cellulose	β-1,4 only	2.02 (5575)	2.6 (13728)	4.7 (35250)	3.3 (30030)
Xyloglucan	β-1,4/6	0.88 (2428)	0.88 (4646)	0.94 (4963)	0.95 (8151)
MCC Avicel PH101	β-1,4 only	0.8 (2152)	0.8 (4418)	0.8 (5920)	0.7 (5920)
PAT Avicel PH101	β-1,4 only	2.0 (5437)	2.5 (13200)	5.0 (36750)	3.1 (26169)
Curdlan	β-1,3 only	0.39 (1076)	0.39 (2059)	0.5 (3750)	0.38 (3260)
**Mannans**					
Galactomannan	β-1,4/6	9.2 (25392)	6.3 (33264)	2.09 (15675)	2.2 (18876)
**Xylans**					
Birchwood xylan	β-1,4 only	0.27 (745)	0.29 (1531)	0.25 (1875)	0.23 (1973)

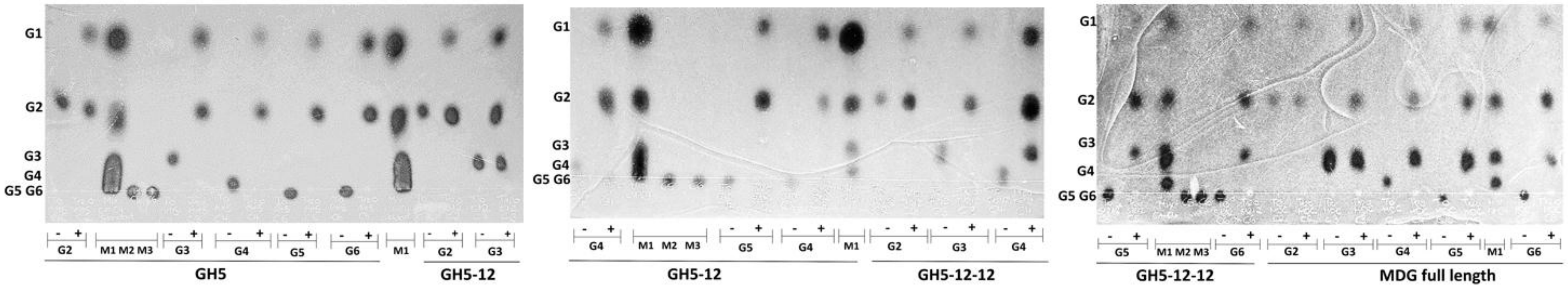

Supplementary Figure 6. Degradation of cellobiose and cellooligosaccharides by the MDG and its truncated versions.
Substrate solutions (1% (w/v)) were mixed with 25 µg of each protein and either directly transferred to ice and the reaction was stopped by the addition of 80 % (v/v) acetone **(-)**, or incubated for 2 h at 60°C **(+)**. Hydrolysis products were separated on aluminum sheet (20 x 20 cm) silica gel 60/kieselguhr F_{254} plates (Merck) using a mixture of ethyl acetate, methanol and H_2O (68:23:9, v/v/v) as mobile phase. Products were visualized with $KMnO_4$ solution (1.5 g $KMnO_4$, 10 g K_2CO_3 and 1.25 ml 10 % aq. NaOH in 200 ml H_2O). G: Number of glucose units. G1 = glucose; G2 = cellobiose; G3 = cellotriose; G4 = cellotetraose; G5 = cellopentaose; G6 = cellohexaose. Marker: M1 = G1,G2,G3,G4. M2 = G5. M3 = G6.

4. Discussion

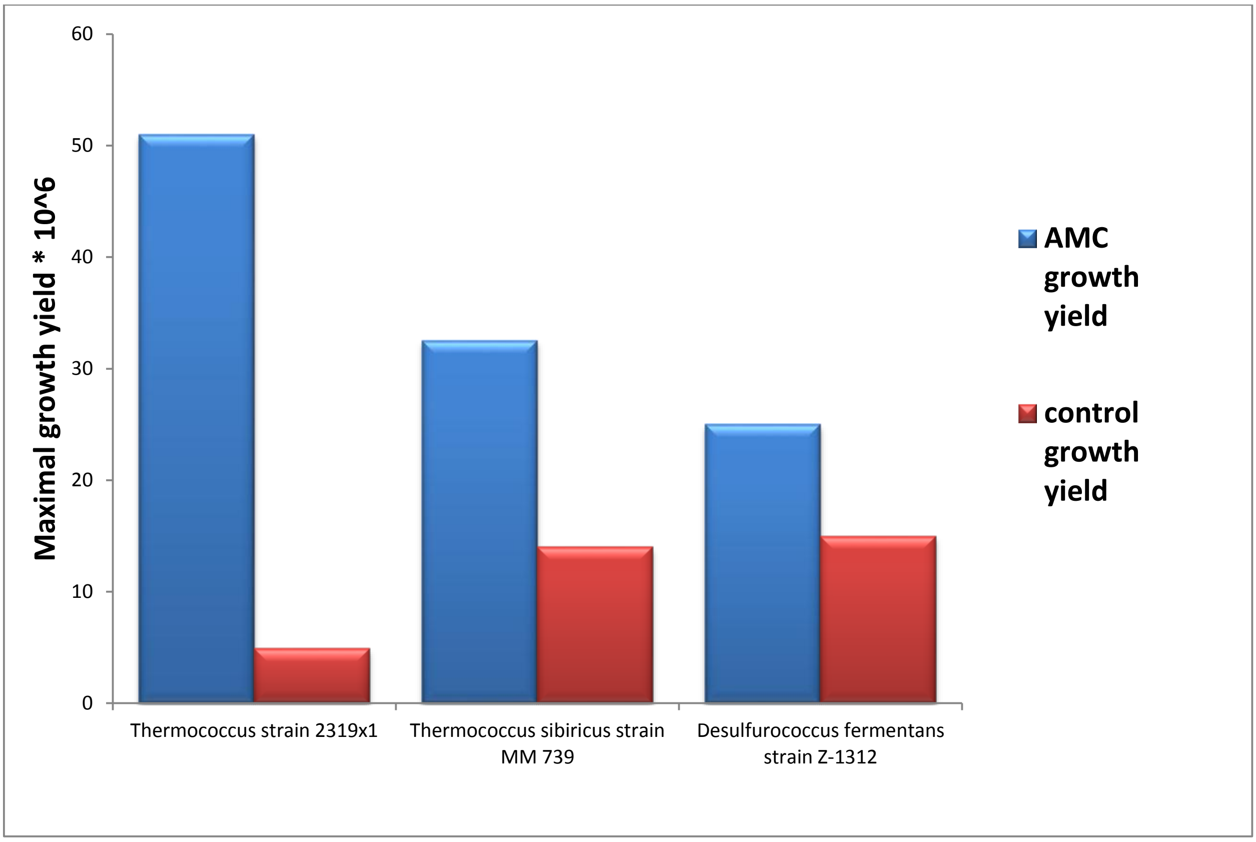

Supplementary Figure 7. Comparative analysis of growth yields of three hyperthermophilic archaeal strains, growing on amorphous cellulose (AMC). All strains were cultivated under their optimal growth conditions. Control means the same medium without AMC substrate.

Supplementary Table 5. Best BLAST hits of CBM2-1 and CBM2-2.

	CBM2-1 (1103-1199)	max	total	% query	% idenity	e-value	acession
1	Cel12E [unidentified prokaryotic organism]	50,4	50,4	0,97	0,37	0,00006	CRL09147.1
2	chitinase [Pyrococcus furiosus]	50,4	50,4	0,97	0,35	0,00007	WP_011012376.1
3	Chain A, Crystal Structure Of Chitin Biding Domain Of Chitinase From Pyrococcus furiosus	46,6	46,6	0,94	0,35	0,0002	2CWR_A
4	chitinase [Thermococcus kodakarensis]	47	47	0,9	0,34	0,0007	WP_048053867.1
5	glycoside hydrolase family 5 [Cyanobacterium aponinum PCC 10605]	47,4	47,4	0,73	0,35	0,0008	AFZ54640.1
6	chitinase [Thermococcus kodakaraensis]	47	47	0,9	0,34	0.001	BAA88380.1
7	hypothetical protein, partial [Thermococcus nautili]	44,7	44,7	0,9	0,29	0.006	WP_042693178.1
8	Chitinase [Thermococcus nautili]	44,7	44,7	0,9	0,29	0.007	AHL22682.1
9	Glycoside Hydrolase Family 5 candidate endoglucanase [Bifidobacterium thermophilum RBL67]	43,9	43,9	0,72	0,35	0.009	AGH41463.1
	**CBM2-2 (1207-1303)**	**max**	**total**	**% query**	**% idenity**	**e-value**	**acession**
1	chitinase [Mycobacterium kansasii]	68,9	135	0,93	0,36	2,00E-11	WP_036448276.1
2	hypothetical protein [Mycobacterium kansasii]	68,9	135	0,93	0,36	4,00E-11	WP_036391652.1
3	hypothetical protein [Mycobacterium kansasii]	68,6	135	0,93	0,36	4,00E-11	WP_042313822.1
4	hypothetical protein [Mycobacterium gastri]	67,4	131	0,93	0,33	1,00E-10	WP_036410849.1
5	hypothetical protein [Mycobacterium kansasii]	66,6	66,6	0,93	0,33	1,00E-10	WP_036444190.1
6	hypothetical protein [Mycobacterium kansasii]	63,2	63,2	0,93	0,34	1,00E-10	WP_042313524.1
7	hypothetical protein MGAST_01715 [Mycobacterium gastri 'Wayne']	67	131	0,93	0,33	2,00E-10	ETW25608.1
8	glycoside hydrolase [Calothrix sp. 336/3]	66,6	66,6	0,91	0,38	2,00E-10	WP_046815061.1
9	cellulose binding domain protein [Mycobacterium kansasii 824]	61,6	61,6	0,89	0,34	3E-09	ETZ98981.1
10	endoglucanase [Microcoleus sp. PCC 7113]	62,8	62,8	0,95	0,32	4E-09	WP_015184298.1
11	hypothetical protein [Mycobacterium kansasii]	62,8	62,8	0,9	0,34	6E-09	WP_023367572.1
12	hypothetical protein [Mycobacterium kansasii]	62,4	62,4	0,9	0,34	6E-09	WP_041327210.1
13	mannan endo-1,4-beta-mannosidase [Streptomyces xiamenensis]	61,2	61,2	0,9	0,37	1E-08	WP_046725132.1
14	chitinase [Mycobacterium kansasii]	61,2	61,2	0,89	0,34	1E-08	KEP39480.1
15	mannan endo-1,4-beta-mannosidase [Streptomyces sp. NRRL F-2890]	61,2	61,2	0,9	0,37	1E-08	WP_030731241.1
16	hypothetical protein MPTA5024_00600 [Microbispora sp. ATCC PTA-5024]	60,8	60,8	0,85	0,35	1E-08	ETK38093.1
17	chitinase [Mycobacterium kansasii]	57,8	57,8	0,9	0,3	1E-08	WP_036394018.1
18	hypothetical protein [Microbispora sp. ATCC PTA-5024]	60,5	60,5	0,88	0,34	2E-08	WP_036322646.1
19	chitinase [Mycobacterium liflandii]	60,8	60,8	0,9	0,31	2E-08	WP_041300546.1
20	hypothetical protein [Calothrix sp. PCC 7103]	60,5	60,5	0,98	0,33	2E-08	WP_035173330.1
21	PE-PGRS family protein [Mycobacterium marinum M]	60,5	118	0,9	0,31	3E-08	ACC42687.1
22	xyloglucanase [Streptomyces griseoflavus]	60,5	60,5	0,95	0,31	3E-08	WP_004922772.1
23	chitinase [Mycobacterium marinum]	60,5	118	0,93	0,31	3E-08	WP_041324889.1
24	hypothetical protein [Mycobacterium gastri]	60,5	60,5	0,93	0,33	4E-08	WP_036414736.1
25	hypothetical protein [Mycobacterium ulcerans]	60,5	117	0,9	0,31	4E-08	WP_011739252.1

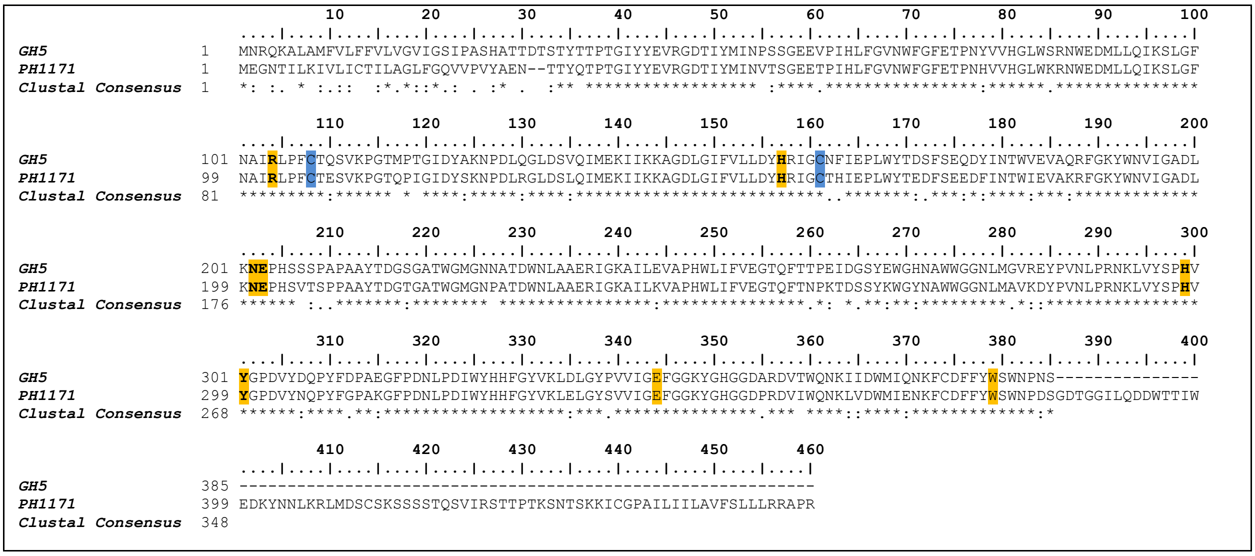

Supplementary Figure 8. Sequence Alignment of the MDG GH5 domain (residues 1 - 385) and the endo-1,4-β-glucanase PH1171 (EGPh) from *Pyrococcus horikoshii* (Ando et al. 2002; Kashima et al. 2005). All identical amino acid residues are marked with a star (*). Conserved residues that are similar to the catalytic cleft of PH1171 (PH1171: Arg102, His155, Asn200, Glu201, His297, Tyr299, Glu342 and Trp377) are highlighted in orange. Cysteins that are involved in the formation of a disulfide bond (PH1171: Cys106 – Cys159) are marked in blue. Alignments were performed with ClustalX and edited with BioEdit.

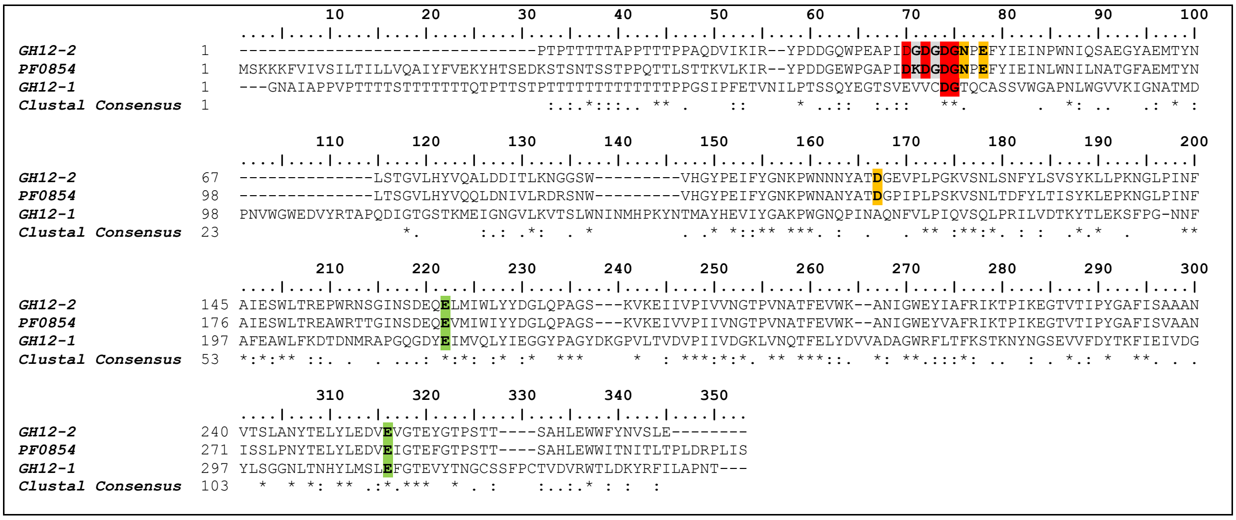

Supplementary Figure 9. Sequence Alignment of the MDG GH12-1 domain (residues 416 - 761), the MDG GH12-2 domain (residues 791 - 1070) (both including upstream linker regions) and the well characterized endo-1,4-β-glucanase PF0854 (EGPf) of *Pyrococcus furiosus* (Bauer et al. 1999). The nucleophile and the proton donor (PF0854: E197, E290, respectively) conserved in family 12 GHs are found in both GH12 domains of the MDG and are highlighted in green. The calcium-binding motif (DxDxDG) as well as the residues involved in metal ion coordination identified in PF0854 (PF0854: Asp68 , Asp70, Asp72, Asn74, Glu76 and Asp142) are found in the upstream linker region of GH12-2 (with low sequence similarity), except Asp142 which is located in the predicted GH12-2 domain. Residues for binding and coordination of Ca^{2+} are highlighted in red and orange, respecively. Alignments were performed with ClustalX and edited with BioEdit.

```
                                  10        20        30        40        50        60        70        80        90       100
                             ....|....|....|....|....|....|....|....|....|....|....|....|....|....|....|....|....|....|....|....|
CBM2-2                   1   ----------NVTIKIDSEWDSGFVVKIYVTNNGNAPVSNWQIKLRMTS--LISGIWGGTYT-VSGDVVTIVPTGSNAVINPGTTIEIGFVASK-QGAYV
1exg_A                   1   --ASSGPAGCQVLWGVN-QWNTGFTANVTVKNTSSAPVDGWTLTFSFPSGQQVTQAWSSTVT-QSGSAVTVRNAPWNGSIPAGGTAQFGFNGSH-TGTNA
CBM2-1                   1   --------G-TLDAKLVNSWGTGAQYEVAVNLDTSS---TWKLLIKIKDG-KISDIWGATIVGTQGDYVVVQPS------SPAASATVGFVTSG-NAPLV
PF1233                   1   GPTTPVPVSGSLEVKVN-DWGSGAEYDVTLNLDGQY---DWTVKVKLAPGATVGSFWSANKQ-EGNGYVIFTPVSWN----KGPTATFGFIVNGPQGDKV
Clustal Consensus        1              :   :  .*.:*   .: :.   .     * : . :     :   *...     .. * .           . :  .**  .   .  .

                                 110
                             ....|....|....|..
CBM2-2                   87  YPELL--GVEIL-----
1exg_A                   96  APTAF--SLNGTPCTVG
CBM2-1                   81  EEAVLLSGDKVLAMWTA
PF1233                   92  EEITLEINGQVI-----
Clustal Consensus        16      :  . :
```

Supplementary Figure 10.Sequence Alignment of the MDG CBM2-1 domain (residues 1103 - 1199), the MDG CBM2-2 domain (residues 1208 - 1303), 1exg_A of *Cellulomonas fimi* and PF1233 of *Pyrococcus furiosus* (Xu et al. 1995; Nakamura et al. 2008; Simpson et al. 2000). Conserved surface-exposed tryptophans in CBM domains are highlighted in orange. The third conserved tryptophan residue is only present in 1exg_A of *Cellulomonas fimi* and PF1233 of *Pyrococcus furiosus*, whereas it is completely missing in the CBM2-1 domain and replaced by a serine in the CBM-2 domain of the MDG (differences are highlighted in grey). Alignments were performed with ClustalX and edited in BioEdit.

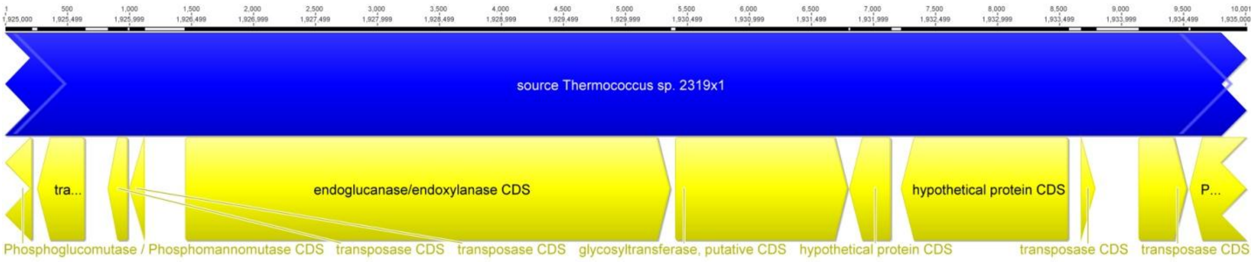

Supplementary Figure 11. The *mdg* gene is encoded in a gene cluster together with a putative glycosyltransferse and two hypothetical proteins in the genome of *Thermococcus* sp. strain 2319x1. The gene cluster is flanked by transposase encoding genes.

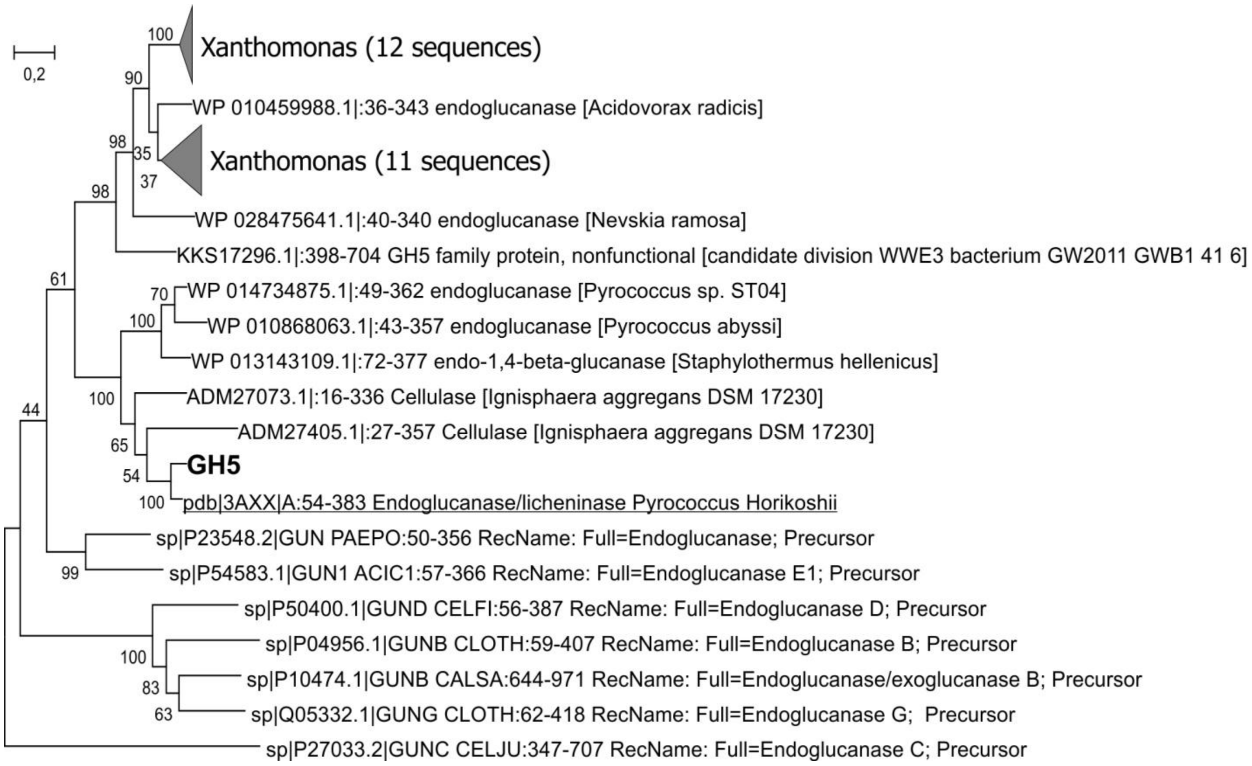

Supplementary Figure 12. Maximum likelihood phylogenetic tree of the GH5 domain of the novel multidomain glycosidase (MDG, ADU37_CDS22600) and the nearest relatives. The tree with the highest log likelihood (-9647.9039) is shown. The number of trees of totally 100 repetitions (bootstrap analysis) in which the associated taxa clustered together is shown next to the branches. The tree is drawn to scale, with branch lengths measured in the number of substitutions per site. The analysis involved 40 amino acid sequences. All positions with less than 95% site coverage were eliminated. There were a total of 292 positions in the final dataset. Evolutionary analyses were conducted in MEGA6 (Tamura et al., 2013). Bar, 20 amino acid substitutions per 100. The *Thermococcus* sp. strain 2319x1 MDG GH5 domain is depicted in bold. The nearest characterized realtive is underlined.

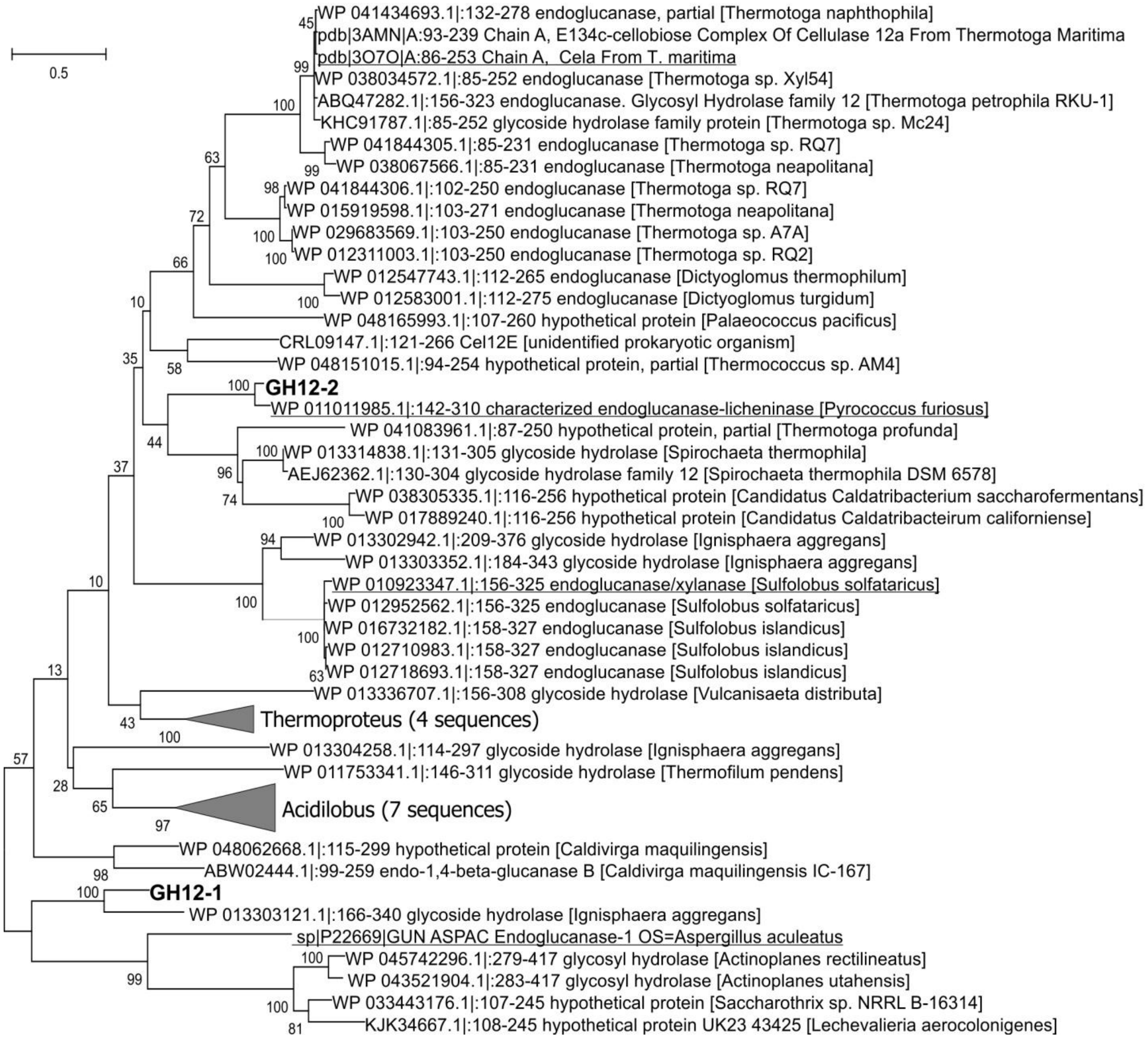

Supplementary Figure 13. Maximum likelihood phylogenetic tree of the GH12 domains of the novel multifunctional, mulitdomain glycosidase (MDG, ADU37_CDS22600) and the nearest relatives. The tree with the highest log likelihood (-9585.6354) is shown. The number of trees of totally 100 repetitions (bootstrap analysis) in which the associated taxa clustered together is shown next to the branches. The tree is drawn to scale, with branch lengths measured in the number of substitutions per site. The analysis involved 54 amino acid sequences. All positions with less than 95% site coverage were eliminated. There were a total of 125 positions in the final dataset. Evolutionary analyses were conducted in MEGA6 (Tamura et al., 2013). Bar, 50 amino acid substitutions per 100. The *Thermococcus* sp. strain 2319x1 MDG GH12-1 and GH12-2 domains are shown in bold. The nearest characterized relatives are underlined.

Supplementary Literature

Tamura K., Stecher G., Peterson D., Filipski A., and Kumar S. (2013). MEGA6: Molecular Evolutionary Genetics Analysis version 6.0. *Molecular Biology and Evolution* 30: 2725-2729.
